# Structure and Dynamics of Macrophage Infectivity Potentiator Proteins from Pathogenic Bacteria and Protozoans Bound to Fluorinated Pipecolic Acid Inhibitors

**DOI:** 10.1021/acs.jmedchem.5c00134

**Published:** 2025-02-20

**Authors:** Victor Hugo Pérez Carrillo, Jacob J. Whittaker, Christoph Wiedemann, Jean-Martin Harder, Theresa Lohr, Anil K. Jamithireddy, Marina Dajka, Benedikt Goretzki, Benesh Joseph, Albert Guskov, Nicholas J. Harmer, Ulrike Holzgrabe, Ute A. Hellmich

**Affiliations:** † Faculty of Chemistry and Earth Sciences, Institute of Organic Chemistry and Macromolecular Chemistry, Friedrich Schiller University Jena, 07743 Jena, Germany; ‡ Groningen Institute for Biomolecular Sciences and Biotechnology, University of Groningen, 9747AG Groningen, The Netherlands; § Institute of Pharmacy and Food Chemistry, University of Würzburg, Am Hubland, 97074 Würzburg, Germany; ∥ Living Systems Institute, University of Exeter, Stocker Road, EX4 4QD Exeter, U.K.; ⊥ Department of Physics, Free University of Berlin, 14195 Berlin, Germany; # Center for Biomolecular Magnetic Resonance, Goethe-University, 60438 Frankfurt/Main, Germany; ∇ Cluster of Excellence “Balance of the Microverse”, Friedrich Schiller University Jena, 07743 Jena, Germany

## Abstract

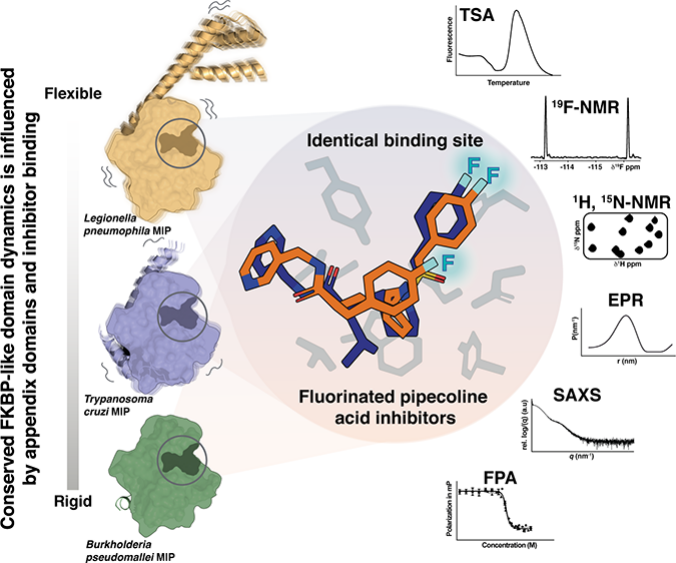

Macrophage infectivity potentiator (MIP) proteins, found in pro- and eukaryotic pathogens, influence microbial virulence, host cell infection, pathogen replication, and dissemination. MIPs share an FKBP (FK506 binding protein)-like prolyl-*cis/trans*-isomerase domain, making them attractive targets for inhibitor development. We determined high-resolution crystal structures of *Burkholderia pseudomallei* and *Trypanosoma cruzi* MIPs in complex with fluorinated pipecolic acid inhibitors. The inhibitor binding profiles in solution were compared across *B. pseudomallei*, *T. cruzi*, and *Legionella pneumophila* MIPs using ^1^H, ^15^N, and ^19^F NMR spectroscopy. Demonstrating the versatility of fluorinated ligands for characterizing inhibitor complexes, ^19^F NMR spectroscopy identified differences in ligand binding dynamics across MIPs. EPR spectroscopy and SAXS further revealed inhibitor-induced global structural changes in homodimeric *L. pneumophila* MIP. This study demonstrates the importance of integrating diverse methods to probe protein dynamics and provides a foundation for optimizing MIP-targeted inhibitors in this structurally conserved yet dynamically variable protein family.

## Introduction

Macrophage infectivity potentiator (MIP) proteins are key microbial virulence factors that facilitate host cell infection, intracellular pathogen replication, and dissemination.^1−4^ They are found in a variety of pathogens of both bacterial and eukaryotic origin, including *Burkholderia pseudomallei*, the causative agent of melioidosis, *Legionella pneumophila*, leading to Legionnaires’ disease,^1,2^ and the protozoan parasites *Trypanosoma cruzi* and *Leishmania* subspecies.^4,5^ These parasites cause Chagas fever in the Americas and global cases of leishmaniasis, diseases classified as neglected tropical diseases (NTDs) by the World Health Organization.^6^ Protozoan NTDs do not only lead to untimely death but also frequently result in disabilities, exacerbating the already severe medical outlook for the patients through an additional socioeconomic toll.^6^

Inhibition of secreted MIP proteins diminishes macrophage invasion and leads to the reduction of overall pathogen load and virulence, as shown for *L. pneumophila* and *B. pseudomallei*, respectively.^2^ Depletion of secreted *T. cruzi* MIP from cell cultures reduces parasite infectivity, an effect that could be rescued by the addition of the *L. pneumophila* MIP homologue,^4,7^ suggesting that the MIP function is at least partially conserved across pathogen species. All MIP proteins share a peptidyl-prolyl-*cis*–*trans*-isomerase (PPIase) domain resembling the FK506 binding protein domains of human FKBPs.^8^ In some cases, MIPs are equipped with additional domains, e.g., dimerization domains in *L. pneumophila* MIP.^9,10^

The high degree of conservation across MIP PPIase domains and their apparent functional redundancy creates an opportunity for the development of pan-MIP inhibitors against a range of pathogens, including those displaying antimicrobial resistance.^11,12^ Considerable progress has been made in identifying and optimizing both natural product-derived and synthetic lead compounds for MIP proteins across diverse pathogens, including *L. pneumophila*, *B. pseudomallei*, and *T. cruzi*, as well as *Neisseria*, *Chlamydia*, and *Klebsiella* species by us and others (see, e.g., refs (11) and (14)). Compounds containing a pipecolic scaffold derived from FK506, the namesake of the FKBP family, demonstrated high effectiveness against both *B. pseudomallei* and *L. pneumophila* MIPs and MIPs from other microbial pathogens, suggesting their potential as a pan-MIP inhibitor.^11−13,15−17^

However, a key gap in the field has remained: the lack of detailed and systematic assessments of the interaction of molecules with pan-inhibitor potential across MIPs from diverse pathogens. A detailed structural and dynamic analysis of MIP homologues from different pathogen species, preferably both of bacterial and protozoan origin, with the same inhibitor scaffold would greatly benefit drug discovery efforts. Such information is currently limited to our study on [4.3.1]-bicyclic sulfonamides^9^ but lacking for pipecolic acid-based inhibitors.

Here, we determined the crystal structures of *B. pseudomallei* MIP (*Bp*MIP), which contains only the core PPIase domain and *T. cruzi* MIP (*Tc*MIP), which features the PPIase domain and a free-standing α-helix (“stalk”), in complex with fluorinated pipecolic acid inhibitors. No crystal structure for *Lp*MIP, which is a dimer composed of a PPIase domain, a stalk helix, and a dimerization domain in each monomer^9^ in complex with the inhibitors, could be obtained. However, we derived a detailed comparison of the different inhibitor binding modes to the various MIP proteins from ^1^H, ^15^N solution NMR spectroscopy. De novo-obtained ^1^H, ^15^N NMR backbone assignments of inhibitor-bound MIP proteins enabled a thorough analysis of MIP protein dynamics in the apo- and ligand-bound states.

Fluorine is found in many drugs and agrochemicals. It is a highly sensitive NMR probe that can be used to study protein–protein and protein–inhibitor complexes.^18−24^ The ^19^F chemical shift can be also used to predict the binding modes of fluorinated ligands and their solvent accessibility.^18,20^ We thus exploited the ^19^F groups of the studied pipecolic acid derivatives to monitor the inhibitor dynamics upon binding to the different MIP proteins. We furthermore made use of the dimeric nature of full-length *Lp*MIP to perform site-specific spin labeling for pulsed electron paramagnetic resonance (EPR) spectroscopy. This, in combination with small-angle X-ray scattering (SAXS), was used to explore the global dynamic consequences of inhibitor binding to dimeric *Lp*MIP.

In this study, we provide the structural basis for the binding of pipecolic acid inhibitors to MIP proteins from diverse microbial pathogens that represent the architectural diversity of the MIP protein family. Despite the high sequence and structural homology across the FKBP-like domain of microbial MIPs, we find differences in their inhibitor binding affinity, inhibition capability, and local inhibitor dynamics that can be used as starting points for future inhibitor optimization. These data further our understanding of the unexpected dynamic variability within a structurally highly conserved protein family and highlight potential challenges for the development of a pan-MIP inhibitor.

## Results

### Closely Related MIP Proteins from Diverse Pathogens Are Inhibited by Pipecolic Acid Derivatives

**Figure 1:**
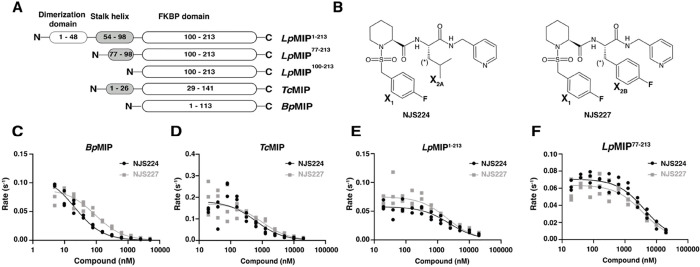
Inhibition of MIP proteins with diverse domain architectures by pipecholic acid inhibitors. (A) Topology of representative MIP proteins from *Legionella pneumophila* (*Lp*MIP), *Trypanosoma cruzi* (*Tc*MIP), and *Burkholderia pseudomallei* (*Bp*MIP) (see also Figure S1). (B) Pipecolic acid derivatives were used as inhibitors for this study. Both NJS224 and NJS227 carry a fluorinated thioaryl group (X_1_) and differ only at the side chain denoted with (*) and contain an iso-butyl (X_2A_) or a *para*-fluorobenzyl (X_2B_) group, respectively.^25^ (C–F) Inhibition of PPIase activity of MIP constructs in the presence of increasing amounts of NJS224 (black) or NJS227 (gray).

To investigate the interaction of pipecolic acid inhibitors with microbial virulence factors, we heterologously expressed and purified the full-length MIP proteins from *L. pneumophila*, *B. pseudomallei*, and *T. cruzi* (Figures 1A,B and S1A–C). With their differences in domain architecture, they represent the architectural variability of the MIP family. All purified constructs showed the expected size, oligomerization state, and secondary structure as gauged by SDS-PAGE, size-exclusion chromatography (SEC), and circular dichroism (CD) spectroscopy (Figure S1D–F).

Peptidyl-prolyl-*cis*/*trans*-isomerase (PPIase) activity for the microbial MIP proteins was determined using a standard coupled assay with chymotrypsin and shown to be inhibited by pipecolic acid derivatives NJS224 and NJS227 (Table 1 and Figure 1).^25^ Two truncated *Lp*MIP constructs, *Lp*MIP^77–213^ and *Lp*MIP^100–213^, that structurally mimic native *Tc*MIP and *Bp*MIP, were also included. *Lp*MIP^77–213^ has typically been used to substitute for the full-length protein in in vitro assays.^26−28^ The shortened constructs are structurally intact (Figure S1); however, truncation resulted in a significant loss of activity. Removal of the dimerization domain (*Lp*MIP^77–213^) reduced the activity by approximately 10% (Table 1). Unexpectedly, truncation to just the PPIase domain (*Lp*MIP^100–213^) reduced activity to below the limit of detection of the experiment (at least 20 times slower than the full-length protein) (Figure S2 and Table 1). This indicates that for *Lp*MIP, the appendage domains beyond the FKBP-like domain play an important functional role.

**Table 1:** Inhibitor Dissociation and Inhibition Constants (*K*_D_, *K*_i_) and Melting Temperatures (*T*_m_) for MIP Proteins in the Absence and Presence of the Inhibitors^a^

construct	PPIase activity (apo)(s^–1^ M^–1^)	*K*_i_ (NJS224) (nM)	*K*_i*,*inhibitor_ (NJS227) (nM)	*K*_D,tracer_ (nM)	*K*_D,inhibitor_(NJS224) (nM)	*K*_D,inhibitor_(NJS227) (nM)	*T*_m_ (apo) (°C)	*T*_m_ (NJS224) (°C)	*T*_m_ (NJS227) (°C)
*B. pseudomallei* MIP	1.3 ± 0.1 × 10^6^	21 ± 2	90 ± 10	52 ± 19	46 ± 7	19 ± 19	61.6 ± 0.1	69.6 ± 0.1	68.5 ± 0.6
*T. cruzi* MIP	3.5 ± 0.1 × 10^4^	600 ± 300	1000 ± 300	856 ± 42	432 ± 125	467 ± 177	47.9 ± 0.7	55.3 ± 0.2	55.0 ± 1.6
*L. pneumophila* MIP^1–213^	1.4 ± 0.1 × 10^5^	2300 ± 400	2000 ± 400	660 ± 160	3990 ± 1970	4700 ± 630	58.8 ± 0.3	62.9 ± 1.1	63.3 ± 0.3
*Lp*MIP^77–213^	1.25 ± 0.09 × 10^5^	4200 ± 500	3700 ± 600	1570 ± 90	3000 ± 710	3300 ± 720	65.1 ± 1.3	66.9 ± 0.9	66.8 ± 0.2
*Lp*MIP^100–213^	<10^4^	nd	nd	48730 ± 1251*	26560 ± 8060^#^	33460 ± 15820^#^	52.6 ± 1.3	54.5 ± 1.1	53.5 ± 0.5

a Of note, for the shortest MIP construct from *L. pneumophila*, *Lp*MIP^100-213^, saturation could not be reached in inhibitor binding experiments (denoted by asterisk in table (*), see also Figure S3) and the protein concentration had to be increased to 10 μM (denoted by a hash sign in table (#); due to the low basal PPIase activity of *Lp*MIP^100-213^, the activity of this construct could not reliably be determined (nd)).

The interaction with two pipecolic acid derivatives was investigated using our recently established fluorescence polarization assay (FPA)^29^ (Table 1 and Figure S3). Here, the displacement of a fluorescent tracer molecule by the prospective inhibitor is monitored. The tracer molecule was designed around a pipecolic acid MIP inhibitor moiety^30^ (Scheme S1) and showed substantial differences in affinity toward the different MIP constructs with dissociation constants ranging from low nM for *Bp*MIP to low μM for *Lp*MIP (Table 1 and Figure S3). Interestingly, *Lp*MIP^77–213^ (*K*_D,Tracer_ = 1570 ± 90 nM) showed a lower affinity than full-length *Lp*MIP^1–213^ (*K*_D,Tracer_ = 660 ± 160 nM). For *Lp*MIP^100–213^, the affinity for the tracer was significantly lower than that for all other constructs, thus requiring a 5-fold increase in the protein concentration. Both NJS224 and NJS227 were able to displace the tracer and yielded displacement *K*_D_ values in the low to medium nM range for *Bp*MIP and *Tc*MIP and in the low μM range for full-length *Lp*MIP and *Lp*MIP^77–213^ (Table 1). The *K*_D,Inhibitor_ values for *Lp*MIP^100–213^ were increased 10-fold compared to the longer *Lp*MIP constructs.

In the PPIase assay, clear inhibitory activity of both NJS224 and NJS227 was observed against every MIP (Table 1 and Figure 1C–F). These compounds were originally developed to inhibit *Bp*MIP^29^ and thus showed strong activity against this protein with *K*_i_ values of 21 ± 2 and 90 ± 10 nM, respectively. Encouragingly, the compounds also showed nanomolar activity against *Tc*MIP (0.6 ± 0.3 and 1.0 ± 0.3 μM, respectively). The activity against *Lp*MIP was lower (2.3 ± 0.4 and 2.0 ± 0.4 μM, respectively). A similar inhibition was observed for truncated *Lp*MIP^77–213^.

To gauge the respective stability of the MIP proteins in the absence and presence of inhibitors, a thermal shift assay was carried out (Figure S4). Interestingly, relatively large differences in the thermal stability between MIP constructs were observed in the absence of inhibitors (Table 1). This included differences in their respective melting temperatures (*T*_m_) of more than 15 and 9 °C, respectively, between constructs of comparable size, i.e., *Tc*MIP/*Lp*MIP^77–213^ (*T*_m_ = 47.9 ± 0.7 and 65.1 ± 1.3 °C, respectively) and *Bp*MIP/*Lp*MIP^100–213^ (61.6 ± 0.1 and 52.6 ± 1.3 °C). This suggests that thermal stability is not solely an intrinsic feature of the PPIase domain or its interdomain contacts but rather an individual property of each MIP that is not readily deduced from the domain architecture. Mirroring the differences in *K*_D,Inhibitor_ values, differences in the net increase in the *T*_m_ values in the presence of inhibitor were observed across MIP constructs, with *Lp*MIP^100–213^ displaying the lowest net gain in *T*_m_ upon inhibitor addition (Table 1 and Figure S4).

### Structural Basis of Inhibitor Binding to MIP Proteins from Pro- and Eukaryotic Pathogens

**Figure 2:**
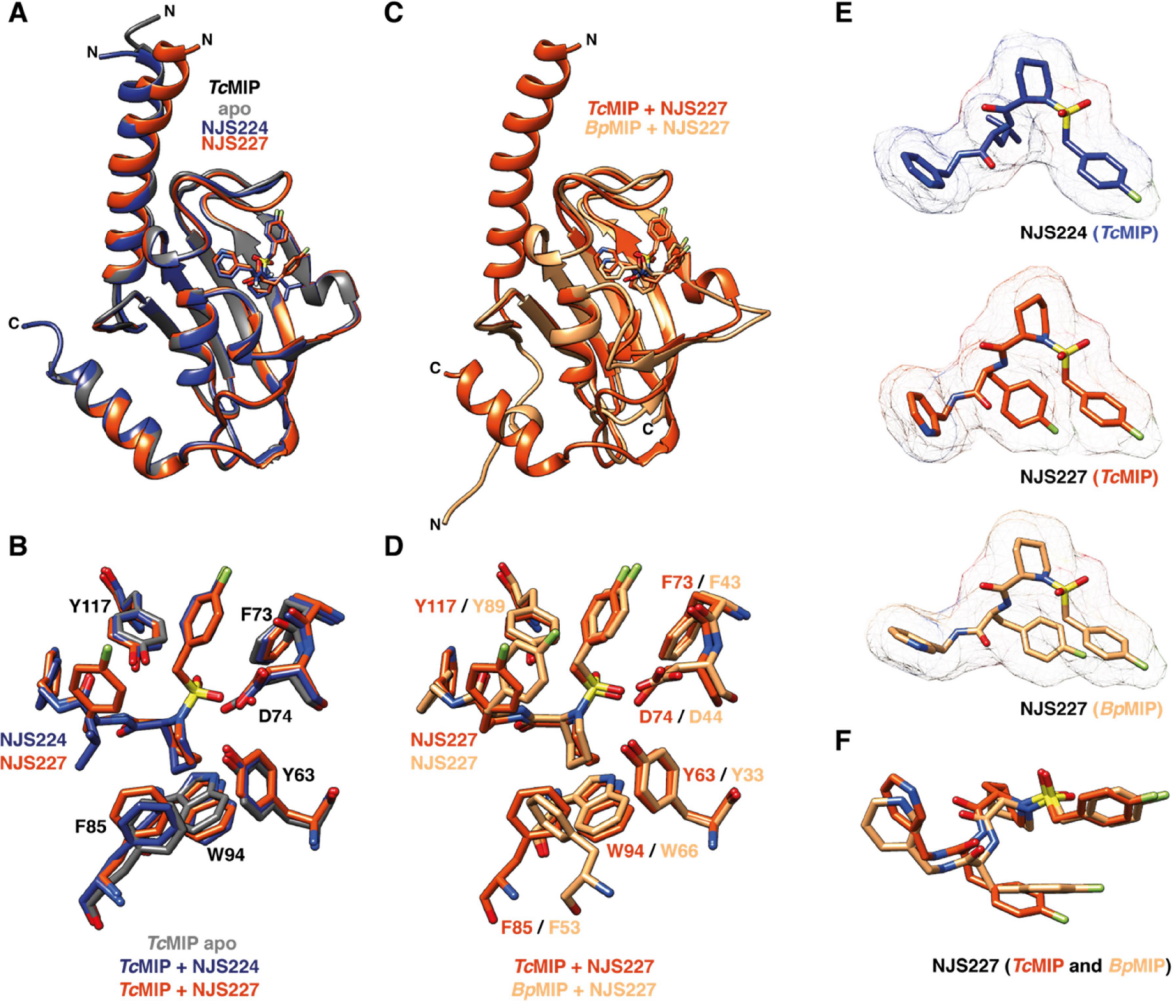
Structures of pipecolic acid inhibitor-bound *Trypanosoma cruzi* MIPand *Burkholderia pseudomalle* MIP. (A) Overlay of X-ray structures of *Tc*MIP in the apo state (PDB: 1JVW, gray) and in complex with NJS224 (PDB: 8P3D, blue) and NJS227 (PDB: 8P42, orange) at 1.70, 1.71, and 2.64 Å resolution, respectively. (B) Zoom into the active site of *Tc*MIP apo (PDB: 1JVW, gray) bound to NJS224 (PDB: 8P3D, dark blue) and NJS227 (PDB: 8P42 orange). (C) Overlay of X-ray structures of *Bp*MIP with NJS227 (PDB: 8P3C, sand) at 2.02 Å resolution and the structure of *Tc*MIP bound to NJS227 (PDB: 8P3D, orange). (D) Zoom into the NJS227-bound active site of *Bp*MIP (PDB: 8P3C, sand) and *Tc*MIP (PDB: 8P3D, orange). (E) Inhibitor molecules in the cocrystal structures of *Tc*MIP and *Bp*MIP can be unambiguously placed in the 2Fo–Fc electron density map. (F) Overlay of NJS227 inhibitors bound to *Tc*MIP (orange) and *Bp*MIP (sand) highlights the difference in the orientation of the fluorine substituents. Atom color code: N—blue, O—red, S—yellow, and F—green.

**Figure 3:**
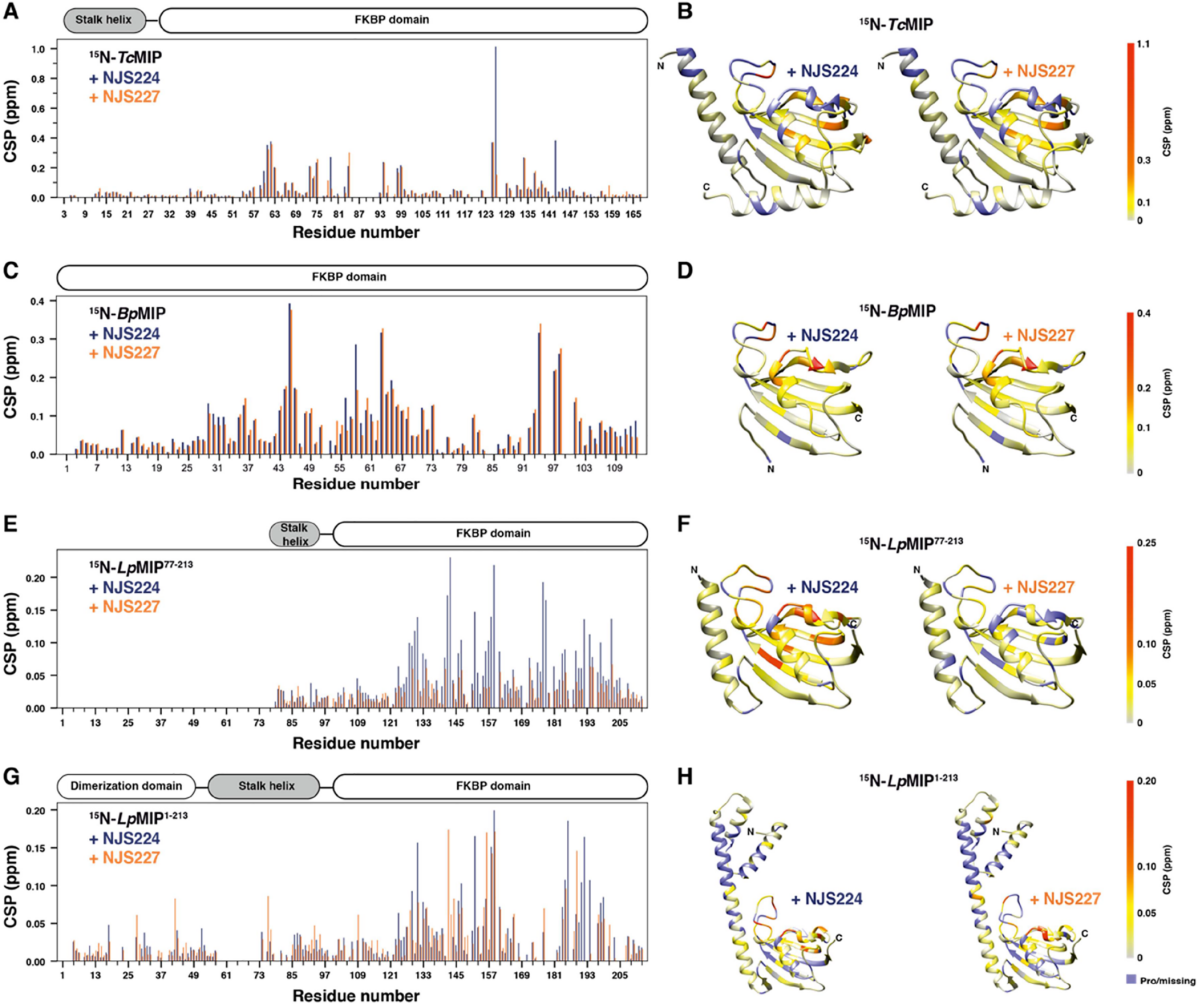
Interaction of pipecolic acid inhibitors with MIP proteins in solution determined by solution NMR. For full ^1^H, ^15^N-HSQC spectra and assignments, see Figures S6–S8. (A, B) CSP between apo- and inhibitor-bound states of *Bp*MIP with pipecolic acid inhibitors (A) and mapped onto the *Bp*MIP X-ray structure (PDB: 8P3C) (B). (C, D) CSP between the apo- and inhibitor-bound state of *Tc*MIP (C) and mapped onto the crystal structure (PDB: 8P3D) (D). (E–H) CSP between apo- and inhibitor-bound states of *Lp*MIP constructs (E, G) and mapped on the respective crystal structures (F, H) (PDBs: 8BKS, 8BJC). For simplicity, only one monomer is shown for full-length *Lp*MIP (H). Proline or unassigned and missing residues in the backbone amide spectra are colored purple for CSP mapped on the respective protein structure.

To elucidate the structural details of the interaction of MIP proteins with their pipecolic acid inhibitors, we used X-ray crystallography and ^1^H, ^15^N solution NMR spectroscopy (Figures 2, 3, and S5). To obtain the backbone NMR assignments of *Tc*MIP, *Bp*MIP, and *Lp*MIP^77–213^ bound to the inhibitors, inhibitor-bound spectra had to be assigned de novo due to the high affinity of the ligands, which gives rise to a new set of peaks in slow exchange (Figures S6 and S7). In the case of *Lp*MIP^1–213^, we could use our previously determined backbone assignments and assignments available from the BMRB (entry 7021)^31^ and obtain the chemical shift assignments of the NJS-inhibitor-bound states through monitoring the chemical shift perturbations (CSPs) upon titration (Figure S8). Because of the poor activity and binding behavior of *Lp*MIP^100–213^ and its reduced stability (Figures 1, S4, and Table 1), it was not included for further structural analyses. All MIP and FKBP proteins share a general domain topology in their core PPIase domain with an amphipathic five-stranded β-sheet wrapping around a short α-helix, thereby forming a hydrophobic active site^32^ (Figure S1). In all cases, we found that the inhibitor molecules bound within the expected FKBP-like domain cleft that forms the MIP active site (Figure 2, Figure 3).

For *Tc*MIP, X-ray crystal structures with both NJS224 and NJS227 were obtained at 1.71 Å (PDB: 8P3D) and 2.64 Å (PDB: 8P42) resolution, respectively (Figures 2, S5A,B, and Table S1). In the two complex structures, both the ligands and the residues lining the binding pocket align almost perfectly (Figure 2B), with an all-atom RMSD of 0.42 Å between the two structures and RMSDs of 0.3 and 0.42 Å compared to the *Tc*MIP apo state structure (PDB: 1JVW). In agreement with very similar *K*_D_ values for either ligand, the distinguishing moieties, i.e., the iso-propyl group in NJS224 and the *para*-fluorobenzyl moiety in NJS227, do not make substantial protein contacts and furthermore face in opposite directions. A highly similar interaction mode for both inhibitors is also apparent from their nearly identical CSP pattern when bound to ^15^N-labeled *Tc*MIP (Figure 3A,B).

*Bp*MIP in complex with NJS227 was resolved to a 2.02 Å resolution (PDB: 8P3C) (Figures 2C, S5C, and Table S1); however, no complex structure with NJS224 could be obtained. Comparing the crystal structures of *Tc*MIP and *Bp*MIP with NJS227, the inhibitor adopts a highly similar binding stance and the respective protein side chains align well (Figure 2F, RMSD = 0.58 Å). In agreement with a similar binding pose for both inhibitors, the CSP pattern of ^15^N-labeled *Bp*MIP titrated with either NJS224 or NJS227 was nearly identical (Figure 3C,D).

The fluorobenzyl group attached to the sulfoxyl group, i.e., the group present in either inhibitor, is nestled into a hydrophobic pocket formed by F43 in β-strand 3a and V97/I98 in the loop between β4 and β5 in *Bp*MIP and the corresponding residues F73 and M125/I126 in *Tc*MIP. This leads to near perfectly superimposable fluorobenzyl moieties across all crystallized inhibitor complexes (Figure 2B–F).

In the crystal structure of *Bp*MIP in complex with NJS227, the sulfoxyl moiety oxygens are 3.5 and 3.3 Å apart from the oxygen atoms of the side chains of D434 in β3a and Y88 in the β4/β5 loop. In *Tc*MIP and *Lp*MIP, the respective positions are occupied by D74/Y117 and D142/Y185, respectively. This agrees with these residues also showing large CSPs upon inhibitor binding in NMR titrations.

In the crystal structures, the inhibitor’s piperidine ring rests within a hydrophobic cage formed by conserved aromatic residues (Y33/Y63, F53/F85, and W66/W94 in *Bp*MIP and *Tc*MIP, respectively). This agrees with the CSP pattern observed for the homologous amino acids in *Bp*MIP, *Tc*MIP, and *Lp*MIP upon titration with either inhibitor (Figure 3). Of note, we previously determined a cocrystal structure of *Lp*MIP with a [4.3.1] bicyclic inhibitor and found it to also engage with a hydrophobic cavity formed by *Lp*MIP residues Y131, F153, and W162.^9^

Together, these data show that the binding poses of the two inhibitors are highly similar for all investigated MIP constructs and that the altered side chain in the inhibitor, i.e., switching from an isopropyl group in NJS224 to a second fluorinated benzenesulfonyl group in NJS227, has no major structural implications for the complexed protein.

However, note that there are important differences between full-length *Lp*MIP and *Lp*MIP^77–213^. Overall, both inhibitors affect the same residues in *Lp*MIP^77–213^; however, the interaction with NJS227 leads to much less pronounced chemical shift changes (Figure 3E). Rather, this inhibitor induces line broadening in the substrate binding pocket, e.g., in residues D142, S143, F153, V158, I159, W162, and G192 (Figure S7B). For full-length *Lp*MIP, differences between the inhibitors are much less pronounced (Figure 3G).

A notable difference between the two inhibitors and the longer and shorter *Lp*MIP constructs is seen for the loop between β-strand 3b and α-helix α1. In *Bp*MIP and *Tc*MIP, hydrophobic residues in this region (V62/I63 in *Bp*MIP and V90/I91 in *Tc*MIP) interact with the inhibitor pyridine group. In *Lp*MIP, the corresponding residues are V158 and I159, whose peaks show line broadening in *Lp*MIP^77–213^ after the addition of NJS227 and CSPs after the addition of NJS224. In contrast, in V158 and I159 in full-length *Lp*MIP, both inhibitors induce chemical shift changes.

In full-length *Lp*MIP, inhibitor binding to the FKBP-like domain not only affects the ligand binding site but is also sensed by residues in the C-terminal region, the stalk helix, and even the dimerization domain. Here, severe line broadening is induced by both NJS224 and NJS227 (Figures 3G and S8). This observation prompted us to investigate the global dynamics of full-length *Lp*MIP in more detail by using EPR spectroscopy and SAXS (see below).

### Local Inhibitor-Induced Perturbations and Dynamic Changes

**Figure 4:**
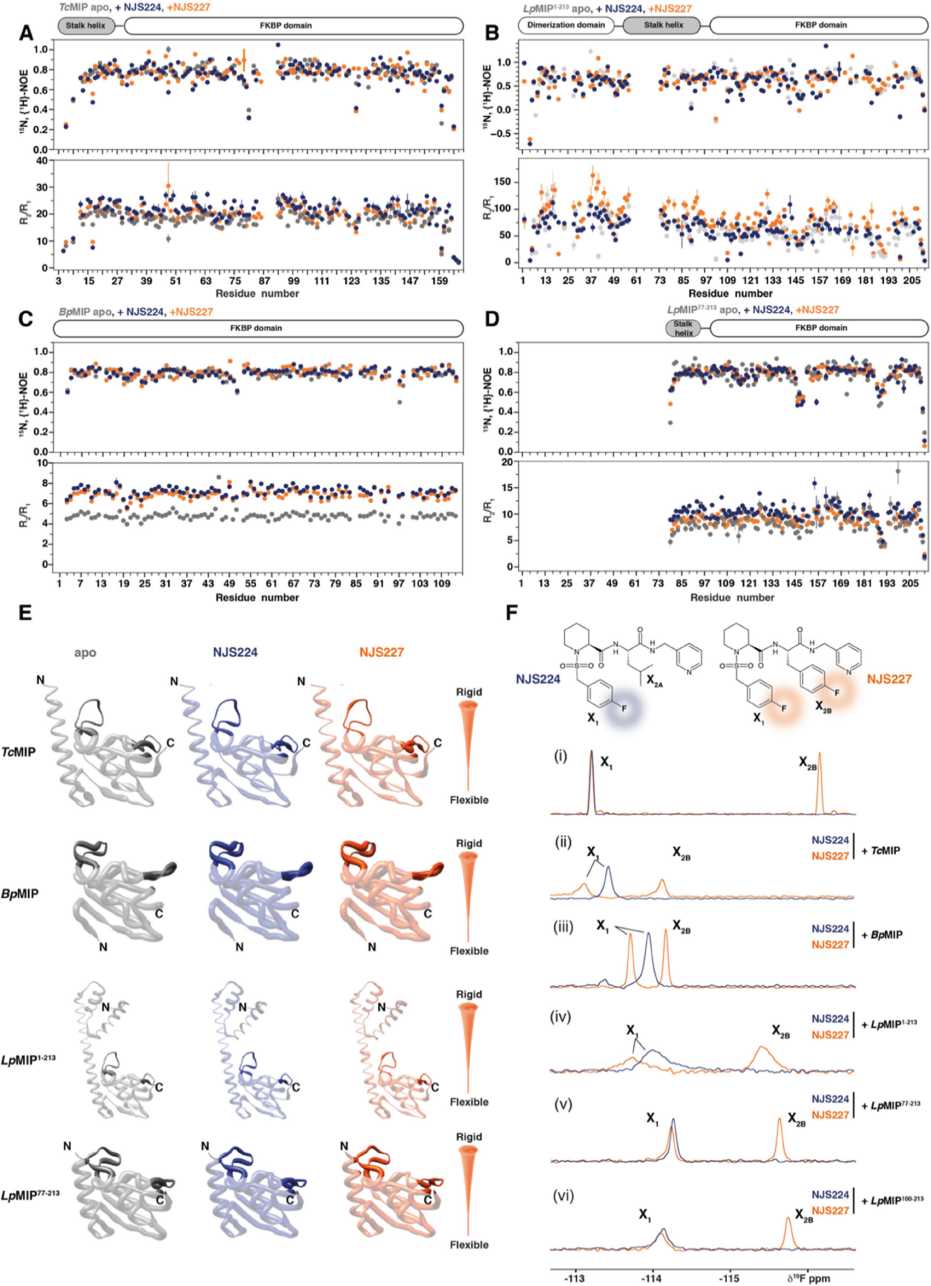
Fast protein backbone dynamics and inhibitor interaction probed by ^1^H, ^15^N, and^19^F NMR. (A–D) ^15^N,{^1^H}-NOE (upper panel) and *R*_2_/*R*_1_ (lower panel) relaxation measurements of (A) *Bp*MIP, (B) *Tc*MIP, (C) full-length *Lp*MIP^1–213^, and (D) *Lp*MIP^77–213^ in the absence (gray) or presence of 5-fold molar excess of NJS224 (blue) and NJS227 (orange). (E) ^15^N,{^1^H}-NOE values plotted onto the structures of *Tc*MIP (PDB: 8P3D), *Bp*MIP (PDB: 8P3C), full-length *Lp*MIP^1–213^ (PDB: 8BJC), and *Lp*MIP^77–213^ (PDB: 8BKS), highlighting the dynamics on the β4/β5 loop (left) and the β3/α1 loop (right). (F) Structures of NJS224 and NJS227 with fluorine moieties highlighted in blue (X_1_) and orange (X_1_ and X_2B_), respectively. (i–vi) ^19^F NMR spectra of the isolated inhibitors (i) and in the presence of purified protein in 5-fold molar excess (ii–vi).

To investigate the consequences of inhibitor binding to microbial MIP proteins in more detail, we investigated the fast backbone dynamics of the proteins using ^15^N,{^1^H}-NOE measurements and correlated protein dynamics measuring the transverse and longitudinal NMR relaxation rates (*R*_2_, *R*_1_) (Figure 4A–E). In addition, we took advantage of the high sensitivity of the fluorine chemical shift and its line width as a reporter for subtle changes in the inhibitor molecule chemical environment and local dynamics^19^ (Figure 4F).

First, we determined the rotation correlation times (τ_c_) for the inhibitor–protein complexes (see the Experimental Section for details). In agreement with our previous findings,^9^ full-length *Lp*MIP is dimeric in both the apo- and inhibitor-bound states, while *Lp*MIP^77–213^, *Bp*MIP, and *Tc*MIP remain monomeric (Table 2).

**Table 2:** Rotation Correlation Time of MIP Proteins^a^

		τ_c_ (ns)			
construct	*M*_w_ (kDa)	theoretical (Stokes–Einstein equation/empirical formula)	apo	+ NJS224	+ NJS227
*Bp*MIP	11.9	5.5/7.2	6.1 ± 0.4	7.6 ± 0.3	7.8 ± 0.3
*Tc*MIP	18.7	8.1/11.5	13.2 ± 1.2	13.8 ± 1.2	14.5 ± 1.3
*Lp*MIP^1–213^	22.9 (monomer)	9.6/14.1 (monomer)	23.9 ± 5.4	27.9 ± 6.2	25.4 ± 4.4
	45.8 (dimer)	17.6/28.3 (dimer)			
*Lp*MIP^77–213^	14.7	6.6/9.0	8.7 ± 1.2	8.8 ± 0.8	9.7 ± 0.8

a Experimental ^15^N *R*_2_/*R*_1_ data for *Bp*MIP, *Tc*MIP, *Lp*MIP^1–213^, and *Lp*MIP^77–213^ in the absence (apo) and presence of NJS224 and NJS227 were used for calculation of rotational correlation time τ_c_ (ns). For comparison, theoretical τ_c_ values approximated from the Stokes–Einstein equation and from an empirical formula are reported. For further details, see the Experimental Section.

Overall, the changes in hetNOE values between the apo- and inhibitor-bound states are very similar for all proteins, as are their respective *R*_2_/*R*_1_ values (Figure 4). This agrees well with a rigid protein core, whose dynamics do not change significantly upon inhibitor binding and with the relatively high melting temperatures of these proteins (Table 1). Importantly, the NMR data also show that the FKBP-like domain of all investigated MIP proteins is relatively rigid throughout, concurring with the observed crystallographic *B*-factors (Figure S5). Our hetNOE data showed two main flexible regions located in the loops between β3 and α1 and β4 and β5 (Figure 4E), and these regions also show the highest B-factors in our crystal structures (Figure S5). Across all MIP proteins, the flexibility of this loop is the most pronounced in *Lp*MIP, while in *Bp*MIP, it showed the most rigidity. It thus seems tempting to speculate that these regions play an important role in the observed differences in inhibitor binding affinity. Interestingly, these loops were also seen to be more flexible in full-length *Lp*MIP compared to *Lp*MIP^77–213^. It thus seems conceivable that the *Lp*MIP stalk helix plays an important role in the dynamics of the β4/β5 loop, which acts as a lid for the substrate. This is an important difference between the construct typically used for *Lp*MIP binding studies, i.e., *Lp*MIP^77–213^ and the native, dimeric *Lp*MIP^1–213^ protein, a finding that may need to be considered in future studies.

Fluorine groups are versatile NMR reporters.^19^ To identify possible differences in inhibitor binding and dynamics, we took advantage of the fluorine moieties within our inhibitor molecules (Figure 4F). To assign the ^19^F resonances, we recorded 1D ^19^F NMR spectra of both molecules in solution (Figure 4F(i)). NJS224 carries a fluorinated thioaryl group (henceforth denoted X_1_) and an isopropyl group (X_2A_) and gives rise to a single resonance at −113.19 ppm. The spectrum of NJS227, which carries the same X_1_ moiety in addition to a *para*-fluorobenzyl group (X_2B_), features two ^19^F resonances. The peak at −113.19 ppm could accordingly be assigned to X_1_, and the resonance at −116.13 ppm could be assigned to X_2B_.

Next, we titrated the fluorinated inhibitors with the purified MIP proteins (Figures 4F(ii–vi) and S9). In agreement with the differences in inhibitor binding affinities, interaction with *Bp*MIP and *Tc*MIP occurs in the slow exchange regime and titration with the three *Lp*MIP constructs shows fast or intermediate exchange (Figure S8).

Even though there is no notable difference in the binding pose of the X_1_ group of either inhibitor bound to *Tc*MIP in the cocrystal structures and although both inhibitors induced near identical chemical shift changes in the ^1^H, ^15^N spectra of *Bp*MIP and *Tc*MIP, the resulting ^19^F chemical shifts and line widths for the X_1_ groups differ in the presence of either protein. This suggests that when bound to the protein, the X_1_ group from either inhibitor experiences slightly different chemical environments and local dynamics (Figure 4E(ii–vii)). Interestingly, for *Bp*MIP, the NJS224 X_1_ line width is broader, while for *Tc*MIP, NJS227 displays broader lines and thus presumably reduced flexibility.

Among the three *Lp*MIP constructs, despite their identical binding pockets and in line with our ^1^H, ^15^N data, the ^19^F chemical shifts of the bound inhibitors were slightly different (Figure 4F(iv–vi)). This shows that the presence of appendage domains must influence the molecular details of binding of the inhibitor to the FKBP-like domain. While the overall increase in line widths for the molecules bound to full-length *Lp*MIP can in part be explained by the larger molecular weight of the dimeric complex, the smallest construct, *Lp*MIP^100–213^, also features line broadening that is more pronounced than in the larger protein *Lp*MIP^77–213^. Furthermore, the chemical shift for X_1_ from NJS224 and NJS227 was identical or near identical when bound to either *Lp*MIP^77–213^ or *Lp*MIP^100–213^ but different when bound to full-length *Lp*MIP. This highlights once more the importance of the appendage domains for binding of the ligand to *Lp*MIP.

Finally, it needs to be noted that for both *Tc*MIP and *Bp*MIP, the X_2B_^19^F resonance showed a much more pronounced CSP than the X_1_ moiety. In contrast, for the *Lp*MIP constructs, both ^19^F peaks showed similar shifts compared with the free inhibitor. This is somewhat unexpected, as X_2B_ makes less protein contacts than X_1_. It is thus possible that the chemical shift differences for X_1_ between the free- and protein-bound form stem mostly from changes in the chemical environment of X_1_ due to protein contacts, while those for X_2B_ are the result of altered intrainhibitor contacts, i.e., a change in the relative orientation of the two fluorinated rings compared to the molecule’s free form.

Overall, the ^19^F NMR data show that fluorine is a convenient reporter to pick up subtle differences in ligand binding dynamics that may remain undetected by X-ray crystallography or protein-observed ^1^H, ^15^N NMR spectroscopy. In addition, the data suggest that despite the high structural similarities between MIP proteins and near identical inhibitor binding poses, the bound inhibitor dynamics can vary across both MIP proteins and closely related compounds.

### Consequences of Pipecolic Acid Inhibitor Binding for the Global Structural Dynamics of Full-Length Dimeric *Legionella pneumophila* MIP

**Figure 5:**
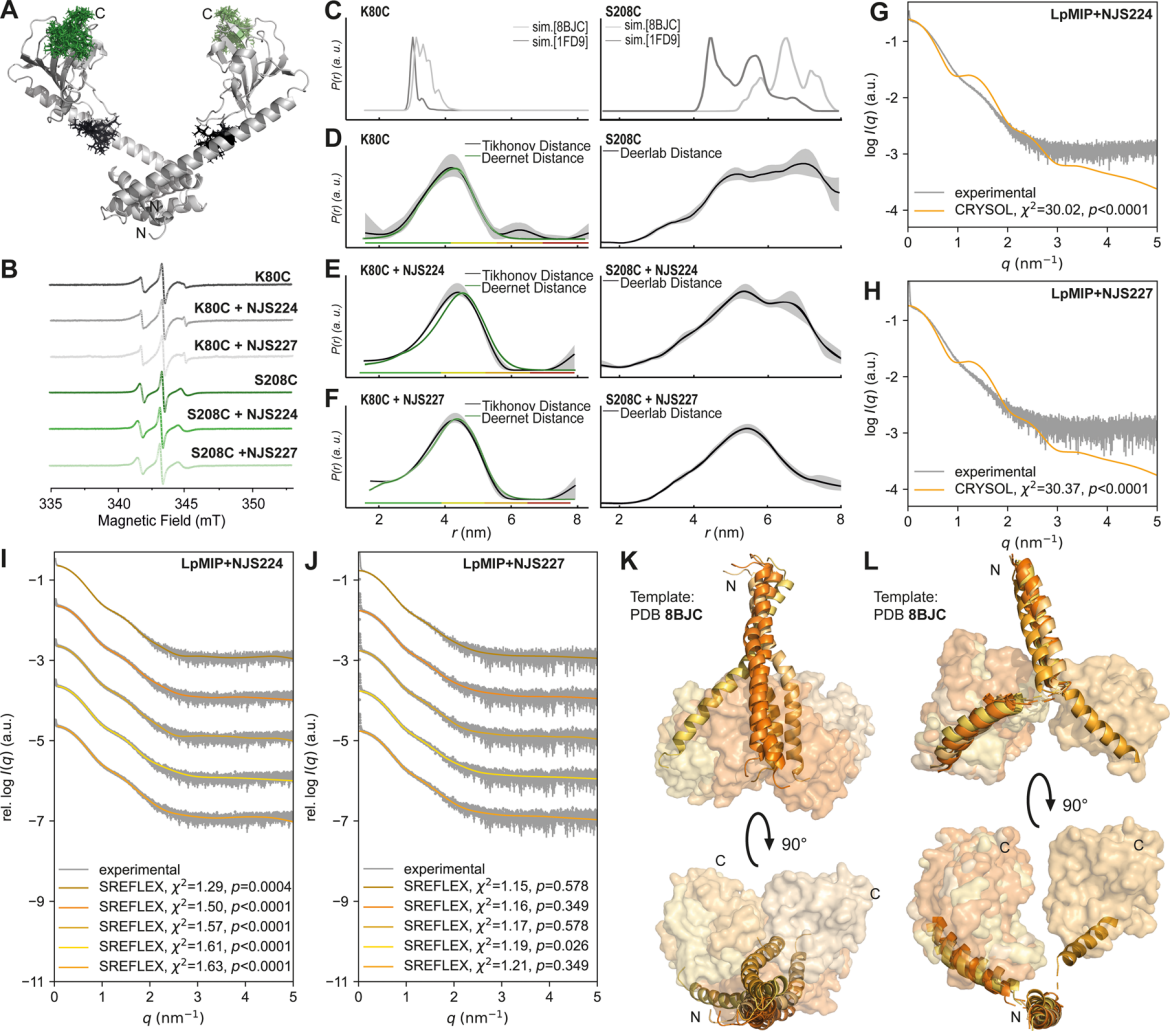
Structural dynamics of full-length, homodimeric *Lp*MIP in solution captured by EPR spectroscopy and SAXS. (A) Protein structure of full-length, homodimeric *Lp*MIP^1–213^ with attached proxy-spin labels on positions K80C (black) and S208C (green). The simulation of the rotamers created with MATLAB-based MMM2022.2 software. (B) Intensity normalized CW EPR spectra of the cysteine variants of *Lp*MIP with the inhibitors NJS224 and NSJ227. (C) Distances between simulated rotamers for *Lp*MIP K80C (left) and *Lp*MIP S208C (right) based on PDB-IDs: 8BJC, 1FD9 with MATLAB-based MMM2022.2 software. (D–F) Distance distributions of the PELDOR/DEER measurements of *Lp*MIP K80C (left) and *Lp*MIP S208C (right), (D) excluding the inhibitors and (E) in the presence of NJS224 and (F) NJS227. Left panel analyzed with Tikhonov regularization and the deep neural networks DEERNet. The rainbow code demonstrates the reliability of the distribution (green shape: width, mean reliable; yellow: width and mean reliable; orange: mean reliable; red: not reliable). Right panel analyzed with the comprehensive Deerlab software for the 5-pulse PELDOR/DEER data. (G, H) Comparison of experimental SAXS profiles of *Lp*MIP in the presence of NJS224 (G) and NJS227 (H) with the computed scattering profile of the apo crystal structure (PDB: 8BJC). The simulated scattering curves were least-squares fitted for 0.5 nm^–1^ < *q* < 1.5 nm^–1^. In both cases, the simulated curves differ significantly from experimental results, suggesting structural changes in solution with the inhibitor. (I, J) Rigid body modeling with SREFLEX was performed to better fit the experimental data. The crystal structure of apo *Lp*MIP (PDB: 8BJC) was used as an input. The calculated scattering profiles of the modeled structures match the experimental data significantly better. (K, L) Rigid body modeling with SREFLEX^31^ suggests global structural changes in NJS224 treated *Lp*MIP (stalk helix shown in cartoon representation, FKBP-like domain in surface representation). The obtained dimer structural models are composed of one monomer with a straight to bent stalk helix (K) and one with a broken helix (L) each. Structural changes in the stalk helices affect the relative position of FKBP-like domains. This results in close contact between the two FKBP-like domains in most of the obtained models. Comparable changes were found for the NJS227-treated sample.

Using protein-detected NMR spectroscopy, we observed that binding of the inhibitor to the FKBP-like domain of full-length *Lp*MIP had long-range consequences for the stalk helix and dimerization domain (Figure 3). Nonetheless, the high molecular weight of homodimeric *Lp*MIP^1–213^ and the unfavorable relaxation behavior hampered a complete analysis of all residues in the stalk helix due to missing resonances by solution NMR. We thus turned to EPR spectroscopy and SAXS to investigate the global dynamics of full-length *Lp*MIP in the presence of the pipecolic inhibitors (Figure 5). These are highly complementary methods that can give insights into the conformational ensemble of proteins and other (bio)macromolecules in cases where X-ray crystallography or cryoelectron microscopy fails to capture the inherent flexibility of a given system.^33−35^

To attach proxyl-spin labels for EPR spectroscopy, we introduced single cysteine mutants at position K80 or S208 in the middle of the stalk helix or the C-terminus of the FKBP-like domain, respectively (Figure 5A). The labeling efficiency was probed with continuous-wave spectroscopy and found to be nearly complete (>85%) for both positions (Figure 5B). Distances between spin label pairs in the *Lp*MIP dimer were measured via pulsed EPR spectroscopy (pulsed electron–electron double resonance (PELDOR, also referred to as DEER). The distances measured for spin labels at either position resulted in a very broad distribution, revealing large protein flexibility. As a comparison, we simulated the possible distances using two previously published crystal structures of apo *Lp*MIP (PDB: 1FD9, 8BJC)^9,10^ (Figure 5C). The experimentally determined distance distributions were seen to be broader than what was obtained from the X-ray structures, thus showing that these structures represent snapshots within the conformational ensemble of the protein.

**Figure 6:**
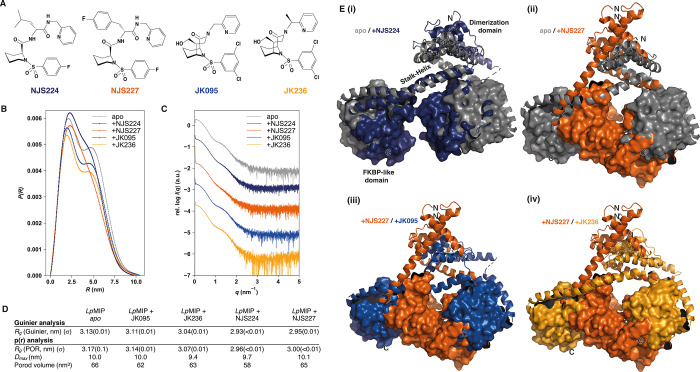
Structural differences of *Lp*MIP^1–213^ in complex with [4.3.1]-aza-bicyclic sulfonamide and pipecolic acid inhibitors. (A) Comparison of different MIP inhibitor structures. Shown are the pipecolic acid derivatives NJS224 and NJS227, as well as the previously investigated bicyclic sulfonamide inhibitors JK095 and JK236.^9^ (B, C) Comparison of experimental SAXS profiles (B) and pair distance distributions (C) of full-length, homodimeric *Lp*MIP^1–213^ in the absence of ligands (gray) or in the presence of inhibitors (colored traces). (D) Results of Guinier and *p*(*r*) analysis show the effects of ligand binding visible in SAXS data analysis. All inhibitors decrease the respective radii of gyration (*R*_g_); however, this effect is more pronounced for the pipecolic acid inhibitor/MIP complexes. The determined Porod volumes show a similar trend upon inhibitor binding. (E) Structural models of the *Lp*MIP/inhibitor complexes. Shown are the top-ranked SREFLEX models.^31^ Dimerization domain and stalk helix are shown as cartoons, with the FKBP-like domain as a surface to highlight the decrease in distance upon inhibitor binding (see labels in (i)). This effect is more pronounced for the pipecolic acid inhibitors than for the [4.3.1]-aza-bicyclic sulfonamides and results in a narrower cleft between the two FKBP-like domains.

In the presence of either NJS224 or NJS227, the overall distance distribution remained wide, in line with continued global flexibility upon inhibitor binding (Figures 5D–F, S10, and S11). However, the addition of NJS227 to *Lp*MIP S208C resulted in a slightly more narrowed distance distribution centered around ∼5.5 nm, which may indicate that this ligand does enable the protein to shift into a slightly more populated state, where the two FKBP-like domains adopt a preferred distance. This is also what we can infer from SAXS (Figures 5G–L and 6), which provides information about the overall shape of a molecule in solution.^34,36,37^

When the scattering profiles of NJS224- and NJS227-bound *Lp*MIP were compared to those we previously reported for apo *Lp*MIP,^9^ the overall dimensions of NJS inhibitor-bound *Lp*MIP were reduced compared to the apo state (Figures 5G,H and 6B). Importantly, the experimental SAXS curves agreed poorly with the theoretical SAXS curves obtained from X-ray structures using CRYSOL (Figure 5G,H) which suggests that the X-ray structures do not, or only partially, capture the conformational dynamics of the protein in solution. Rigid body modeling with SREFLEX^38^ was performed using the *Lp*MIP *apo* crystal structure as a starting point (PDB: 8BJC). The resulting theoretical scattering profiles are shown in Figure 5I,J. When the SREFLEX models included kinking of the stalk helix, thereby reducing the complex dimensions, significantly better fits with the experimentally determined scattering data could be achieved (Figures 5G–J and 6E). This suggests that inhibitor binding induces global *Lp*MIP conformational changes. We previously investigated the interaction of [4.3.1]-aza-bicyclic sulfonamide inhibitors with *Lp*MIP and found that these molecules also affect the conformational dynamics of *Lp*MIP.^9^ However, the pipecolic acid inhibitors investigated here induced stronger conformational changes in line with a closer association of the two FKBP-like domains due to stalk helix kinking (Figure 6). This shows that structurally related inhibitors can evoke distinct conformational signatures on the protein.

## Discussion and Conclusions

While public awareness is currently mostly focused on viral infections, bacterial and protozoan pathogens also claim countless lives each year. In addition, many of the diseases caused by these pathogens can be chronic and severely disabling, thereby placing a tremendous burden on patients and their caregivers, as well as the respective economic and healthcare infrastructure. A unifying factor in many pathogens with an intracellular lifecycle stage is the presence of MIP virulence factors.^39,40^ We thus sought to compare the ability of archetypical microbial MIP proteins to interact with inhibitors and to create a roadmap for the design of novel inhibitors.

Using a combination of biophysical methods, we looked at the interaction of pipecolic acid derivative inhibitors of microbial MIP proteins with bacterial and protozoan pathogens. In line with prior observations for different inhibitor scaffolds, a range of affinities is observed, with *B. pseudomallei* MIP showing very high inhibition constants, followed by *T. cruzi* MIP and *L. pneumophila* MIP. The crystal structures of *Tc*MIP and *Bp*MIP, as well as the NMR-based CSP data for all three proteins, indicate highly similar inhibitor binding poses. The differences in affinity may thus be due to the absence or presence of MIP appendage domains, such as the stalk helix, which allosterically affects the ligand binding site in the FKBP-like domain or the local inhibitor and/or protein dynamics in the complex.

While local dynamics of the protein backbone were only marginally impacted by the inhibitor interaction, we observed that global conformational dynamics is greatly influenced. Interestingly, this effect was stronger for the pipecolic acid inhibitors tested here compared to the bicyclic inhibitors we investigated earlier^9^ (Figure 6). Excitingly, this shows that interactions with chemically distinct ligands can fine-tune the structural dynamics of multidomain MIP proteins and suggests scissor-like motions for dimeric MIP proteins that may lead to transient association of the two FKBP-like domains.

Here, using ^19^F NMR, we demonstrate that despite nearly identical ligand binding sites, the inhibitor interactions and dynamics indeed subtly differ across MIPs. This was the most striking for *Lp*MIP deletion constructs lacking part or the entire appendage domains, which we found to severely impact substrate binding, PPIase activity, inhibitor binding, and protein stability. Thus, *L. pneumophila* MIP appendage domains may play both a structural and a functional role. It remains to be investigated why MIP proteins from other species do not always require these domains. Of note, it was recently reported that *Lp*MIP is not the sole virulence factor in *L. pneumophila* responsible for host macrophage infections.^28^ It is therefore tempting to speculate that for some reason, the intrinsic high ligand binding affinity of, e.g., *Bp*MIP has enabled this protein to circumvent the need for “helper domains” and may probably enable it to act as a highly efficient virulence factor for its host. In contrast, as we have seen, *Lp*MIP requires the presence of additional stabilizing domains to properly carry out its function, and the loss of the appendage domains results in severe protein destabilization.

This may result from the fact that, as our NMR dynamics measurements have shown, the FKBP-like domain of *Lp*MIP feature loops that are intrinsically more flexible than those of the homologous domain from *Bp*MIP and *Tc*MIP. Whether the *Lp*MIP appendages play additional roles in the *Legionella* life cycle remains to be seen. Intriguingly, our ^1^H, ^15^N NMR data on full-length *Lp*MIP showed that the resonances for the residues forming the loop between β3 and α1 in the FKBP-like domain (residues 57–63) were never visible in the spectra. In the shorter *Lp*MIP^77–213^ construct, as well as in *Tc*MIP and *Bp*MIP, this region seems to display more flexibility, thus resulting in sharper line widths. This, together with our observations from SAXS that the FKBP domains of full-length *Lp*MIP can come into close proximity, makes it tempting to speculate whether the observed line broadening is a result of transient FKBP-like domain dimerization. Importantly, our data show that truncation constructs of MIP proteins for functional and inhibition studies should be handled with care and that ^19^F NMR is a straightforward tool to quickly screen possible differences in interaction modes across closely related compounds and proteins.

In summary, significant progress has been made in identifying and optimizing both natural product-derived and synthetic lead compounds for MIP proteins across diverse pathogens; however, a persistent knowledge gap has remained the lack of detailed and systematic assessments of the interaction of these molecules across MIPs from diverse pathogens. Using an integrated structural approach, our work provides comprehensive evidence that differences in the dynamic profiles of MIP proteins—rather than structural variations—play a crucial role in inhibitor interactions. These findings introduce a new perspective on MIP-targeted drug development and have broader implications for designing selective inhibitors for closely related protein families.

## Experimental Section

### Cloning, Protein Expression, and Purification

Genes coding for *Legionella pneumophilaLp*MIP^1–213^, *Lp*MIP^77–213^, *Lp*MIP^100–213^, *Trypanosoma cruzi* TcMIP, and *Burkholderia pseudomalleiBp*MIP (UniProt-KB: Q63J95) were obtained from GenScript (Piscataway Township, NJ, USA). *Lp*MIP^1–213^, *Lp*MIP^77–213^, *Lp*MIP^100–213^, and *Bp*MIP were cloned into the pET-11a vector with an N-terminal His_6_-tag, followed by a TEV cleavage site. *Tc*MIP was cloned into the pET-11a vector with an N-terminal His_6_-tag, followed by a SUMO-tag and a Ulp1 cleavage site. Of note, we started numbering *Legionella pneumophila* MIP (UniProt-KB: Q70YI1) and *Trypanosoma cruzi* MIP (UniProt-KB: Q09734) with residue 1 behind the signal peptide sequence.

Transformation and cell growth were carried out as previously^9^ described. Briefly, freshly transformed *E. coli* BL21 gold (DE3) cells were grown at 37 °C to an OD_600_ between 0.6 and 0.8, then induced with 1 mM IPTG and grown overnight at 20 °C. ^2^H,^15^N-labeled *Lp*MIP was obtained by growing cells in commercially available Silantes OD2 *E. coli* medium (Silantes GmbH, Munich, Germany). ^13^C, ^15^N-labeled *Lp*MIP^77–213^ and *Lp*MIP^100–213^, *Tc*MIP, and *Bp*MIP were obtained by growing cells in minimal medium with ^15^N-NH_4_Cl and ^13^C-glucose as the sole nitrogen and carbon sources. Cells were harvested by centrifugation (6220 × *g*,15 min, 4 °C). Afterward, the cell pellet was frozen in liquid N_2_ and stored at −20 °C until further use.

For protein purification, the cell pellet was dissolved in lysis buffer (20 mM Tris pH 8, 20 mM imidazole pH 8, 300 mM NaCl, 0.1% (v/v) Triton X-100, 1 mM DTT, 1 mM benzamidine, 1 mM PMSF, DNase, RNase, and lysozyme). Cells were disrupted by passing them three times through a microfluidizer (Maximator) at 18,000 psi. Cell lysate was centrifuged at 48,380 × *g*, 30 min, 4 °C, and the resulting supernatant was loaded onto a NiNTA column (Qiagen, Hilden, Germany) previously equilibrated with washing buffer (20 mM Tris pH 8, 300 mM NaCl, and 20 mM imidazole). After washing with 10 CV (column volumes) of washing buffer, the protein of interest was eluted with 5 CV of elution buffer (20 mM Tris at pH 8, 300 mM NaCl and 500 mM imidazole at pH 8). Proteins were dialyzed overnight at 4 °C in 20 mM Tris pH 8 and 300 mM NaCl in the presence of His-tagged TEV protease (1:20 mol/mol) to cleave the His_6_-tag from the MIP constructs.

Dialyzed protein was then loaded onto a fresh NiNTA column. The flow-through was collected, and the column was washed with 4 CV of washing buffer to obtain the maximum amount of tag-free MIP proteins. For the purification of *Lp*MIP^100–213^, all buffers were adjusted to pH 7. After concentration, the proteins were loaded on a size-exclusion column (HiLoad 16/600 Superdex pg, Cytiva, Freiburg, Germany) equilibrated with a size-exclusion buffer (20 mM Tris at pH 7, 150 mM NaCl). The fractions containing pure protein were pooled, and sample purity was verified by SDS-PAGE.

### Synthesis of Inhibitors

NJS224 and 227 were synthesized according to Scheuplein et al.^29^

### PPIase Assay

MIP activity was determined as previously described.^25^ Briefly, rate measurements were performed using a FLUOstar Optima microplate reader (BMG Labtech) kept in a cooled incubator (incu-270C, SciQuip) at 6 °C (giving an instrument working temperature of 8 °C). The substrate peptide succinyl-Ala-Phe-Pro-Phe-4-nitroanilide (Bachem #4016001) was mixed with 35 mM Hepes pH 7.8 to give a final reaction concentration of 150 μM in a 96-well plate (Greiner #655101). For inhibition experiments, either compound was added at 10–20,000 nM (final concentration) in a series of 2-fold dilutions in DMSO (0.5%(v/v) final DMSO concentration). Purified MIP was added to the working concentration with shaking. After 10 s, chymotrypsin (Merck No. C4129) was added to a final concentration of 2.5 mg/mL, followed by 5 s of shaking. Hydrolysis of the substrate was then detected at 390 nm, with readings taken at 1 s intervals until there was no further change in absorbance. Absorbance at 600 nm was measured to determine the background. The pseudo-first-order rate constant was calculated from the difference between 390 and 600 nm reading using GraphPad Prism v 10.2.3 (Dotmatics) using eq 1:
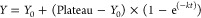
1where *Y* is the measured absorbance, *Y*_0_ is the value of *Y* at *t*_0_, Plateau is the asymptote of *Y*, *k* is the rate constant (s^–1^), and *t* is the time (s). Plateau, *Y*_0_, and *k* were fitted using nonlinear regression. Data were excluded if the fit gave an *R*^2^ value of less than 0.8 as such data represent experiments that have reached a plateau before sufficient data were collected and give unreliable fits.

For determination of *k*_cat_/*K*_M_, the observed rate was plotted against enzyme concentration, with the gradient fitted by linear regression representing *k*_cat_/*K*_M_*.*^41^ For determination of *K*_i_, the modified eq 2 was used^42^ for fitting using nonlinear regression:
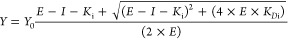
2where *Y* is the measured rate, *Y*_0_ is the measured rate with no inhibitor, *E* is the enzyme concentration, and *I* is the inhibitor concentration. *E* was set to the enzyme concentration used, and *Y*_0_ and *K*_i_ were fitted using nonlinear regression.

### Circular Dichroism Spectroscopy

CD measurements were conducted on a Jasco J-1500 CD spectrometer (Jasco, Gross-Umstadt, Germany) with 1 mm quartz cuvettes using 3.5 μM purified protein in 5 mM Tris at pH 7 and 2.5 mM NaCl. Spectra were recorded at 25 °C in a spectral range between 190 and 260 nm with 1 nm scanning intervals, 1 nm bandwidth, and 50 nm/min scanning speed. All spectra were obtained from automatic averaging of five measurements.

### Thermal Stability Assay

Ten micrograms of purified *Lp*MIP and *Tc*MIP constructs in 20 mM Tris pH 7 and 150 mM NaCl were incubated with a final concentration of 0.02% (v/v) DMSO or a 5-fold molar excess of NJS224 and NJS227 in DMSO (0.02% (v/v) final concentration). A 2.5 μL portion of a 50× SYPRO Orange (Merck) stock was added to each sample directly before measurement of the melting temperature in a 96-well plate on a QuantStudio 1 Real-Time PCR System reader (Thermo Fisher) with a temperature increase of 0.05 °C/s. The same protocol was followed for *Bp*MIP but using a concentration of 25 μg of protein and a concentration of 10× of SYPRO Orange. The fluorescence of SYPRO Orange was measured using the filter calibrated for SYBR GREEN with an excitation filter of 470 ± 15 nm and an emission filter of 520 ± 15 nm.

### Fluorescence Polarization Assay

The binding affinities of the MIP inhibitors for the respective MIP proteins were determined using fluorescence polarization according to the same procedures as described previously.^29,30^

Initially, the compound NJS254, labeled with fluorescein, was titrated with the MIP proteins/constructs. This results in the dissociation constant *K*_D_ for the respective target. Furthermore, *K*_D_values can be calculated by displacement of this tracer from the tracer–protein complex by the inhibitors. NJS254 (see the SI) and all other compounds were prepared in a DMSO stock solution and then diluted with the assay buffer (20 mM HEPES, 0.002% (v/v) Triton X-100, 13.4 mM KCl). NJS254 dilutions were performed to a final concentration of 10 nM, which is four times higher than the final concentration in the well. All inhibitors were prepared in three individual dilution series (300 μM–0.03 nM). Subsequently, 15 μL each (of the compound and tracer) was mixed with 30 μL of protein solution in black 384-well plates (Greiner Bio-One, Kremsmünster, Austria, #781900). The protein concentration is based on the affinity to the tracer to obtain a sufficient dynamic range (ΔmP). The final concentration in the well was 250 nM for *Bp*MIP and 2 μM for *Tc*MIP, *Lp*MIP^77–213^, and *Lp*MIP^1–213^, whereas 10 μM had to be used for *Lp*MIP^100–213^. After incubation for 30 min in the dark at room temperature, fluorescence polarization was measured (Mithras LB 940, Berthold Technologies, Bad Wildbad, Germany), and competition curves were analyzed by using GraphPad Prism 8.0.1.

### Crystallization, Data Collection, and Structure Determination

Following SEC, each of the proteins was kept in a solution of 20 mM Tris and 150 mM NaCl at pH 7.0 and concentrated to 15 mg/mL using a 10,000 MWCO concentrator. Each protein was mixed with the crystallization buffer in a ratio of 2:1, respectively. Crystals of *Tc*MIP NJS224 and NJS227 were obtained using sitting drop vapor diffusion via the Molecular Dimensions SG1 (Shotgun) screening kit in the following conditions: *Tc*MIP NJS224 0.2 M magnesium chloride hexahydrate and 0.1 M Bis–Tris, pH 6.5, 25% (w/v) PEG 3350; *Tc*MIP NJS227 0.2 M sodium acetate trihydrate, 0.1 M sodium cacodylate, pH 6.5, 18%(w/v) PEG 8000. Crystals of His-tagged *Bp*MIP NJS227 were obtained via a custom screening kit in the following conditions: 1.2 M ammonium sulfate, 0.1 M Bis–Tris, pH 5.5, 17%(w/v) PEG 400. All crystals were briefly soaked in 30% (v/v) glycerol for cryoprotection and subsequently flash-frozen in liquid nitrogen in preparation for diffraction experiments at synchrotron energy. Data were collected at beamline ID23-1 (ESRF, Grenoble). Crystals of *Tc*MIP and *Bp*MIP diffracted between 1.7 and 2.6 Å resolution (Table S1). Data were processed by XDS, and structures were solved by Molecular Replacement with Phaser^43^ using previously published models of MIPs (PDB ID: 1JVW, 2KE0).^7,44^ Manual rebuilding was performed with COOT^45^ and refinement with Refmac.^46^ The refined models were deposited into the PDB repository with the IDs 8P3D, 8P42, and 8P3C. Images were prepared using Pymol (Schrödinger, LLC) and CorelDRAW (Corel).

### NMR Spectroscopy

All NMR spectra were recorded on a 600 MHz Bruker Avance III HD or Neo NMR spectrometer system equipped with 5 mm triple resonance cryoprobes. D_2_O was used for field frequency locking. The sample temperature was set to 298.2 K. The ^1^H chemical shifts of the ^13^C, ^15^N-labeled *Bp*MIP, ^13^C, ^15^N-labeled *Tc*MIP, ^13^C, ^15^N-labeled *Lp*MIP^77–213^, and ^2^H, ^15^N-labeled *Lp*MIP^1–213^ were directly referenced to 3-(trimethylsilyl)propane-1-sulfonate (DSS). Indirect ^13^C and ^15^N chemical shift referencing was applied to the ^1^H DSS standard by the magnetogyric ratio. *Lp*MIP^1–213^ was measured in 50 mM Tris HCl pH 7, 150 mM NaCl, 0.1 mM DSS, 0.05% NaN_3_, and 10% D_2_O. Sample conditions for *Bp*MIP, *Tc*MIP, and *Lp*MIP^77–213^ were the same except 20 mM Tris HCl, pH 7, was used. Final protein concentrations were in the range of 100 μM. All spectra were processed using Bruker Topspin 4.3.0 and analyzed using CcpNmr Analysis v2.5^47^ within the NMRbox^48^ virtual environment.

NMR backbone assignments of *Bp*MIP (BMRB entries 16,406 and 17,151), *Tc*MIP (BMRB entry 27,531), *Lp*MIP^1–213^ (BMRB entry 7021), and *Lp*MIP^77–213^ (BMRB entry 6334) are available in Biological Magnetic Resonance Data Bank and were transferred to our spectra. Band-selective excitation short-transient (BEST) transverse relaxation-optimized spectroscopy (TROSY)-based HNCA experiments under our buffer conditions and in the presence of ligands NJS224 and NJS227 were recorded for assignment verification.

Longitudinal and transverse ^15^N relaxation rates (*R*_1_ and *R*_2_), as well as ^15^N-{^1^H} steady-state nuclear Overhauser effect (^15^N,{^1^H}-NOE) values, were measured by employing standard NMR pulse sequences implemented in the Bruker Topspin library. TROSY-sampling pulse sequences were used for *Lp*MIP^1–213^ due to the high molecular weight to ensure high data quality. ^15^N *R*_1_ and *R*_2_ relaxation rates of the ^15^N–^1^H bond vectors of backbone amide groups were extracted from signal intensities (*I*) by a single exponential fit according to eq 3:
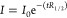
3

The variable relaxation delay *t* was set to 1000, 20, 1500, 60, 3000, 100, 800, 200, 40, 400, 80, and 600 ms in the *R*_1_ relaxation experiments of *Bp*MIP, *Tc*MIP, and *Lp*MIP^77–213^. For *R*_1_ measurements of *Lp*MIP^1–213^, the variable relaxation delay *t* was set to 1000, 5000, 1500, 60, 3000, 100, 800, 200, 40, 400, 80, and 600 ms. In all *R*_2_ relaxation experiments, the variable loop count was set to 36, 15, 2, 12, 4, 22, 8, 28, 6, 10, 1, and 18. The length of one loop count was 16.96 ms. In the TROSY-based *R*_2_ experiments, the loop count length was 8.48 ms, and the first loop count was set to 3 instead of 36. The variable relaxation delay *t* in *R*_2_ experiments is calculated by the length of one loop count times the number of loop counts. The interscan delay for the *R*_1_ and *R*_2_ experiments was set to 5 s.

The ^15^N-{^1^H} steady-state nuclear Overhauser effect measurements (^15^N,{^1^H}-NOE) were obtained from separate 2D ^1^H–^15^N spectra acquired with and without continuous ^1^H saturation, respectively. The ^15^N,{^1^H}-NOE values were determined by taking the ratio of peak volumes from the two spectra, ^15^N,{^1^H}-NOE = *I*_sat_/*I*_0_, where *I*_sat_ and *I*_0_ are the peak intensities with and without ^1^H saturation. The saturation period was approximately 5/*R*_1_ for the amide protons.

The averaged ^1^H- and ^15^N-weighted CSP observed in ^1^H,^15^N-HSQC spectra was calculated according to eq 4:
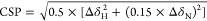
4where Δδ_H_ is the ^1^H chemical shift difference, Δδ_N_ is the ^15^N chemical shift difference, and CSP is the averaged ^1^H- and ^15^N-weighted chemical shift difference in ppm.

The oligomerization state of a protein can be estimated from the rotational correlation time (τ_c_), the time it takes the protein to rotate by one radian under Brownian rotation diffusion. Under the assumption of a spherical globular protein and isotropic motion, τ_c_ (in ns) can be roughly approximated from the Stokes–Einstein eq 5:
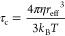
5where η is the viscosity (0.89 mPa·s for water at 298.2 K), *k*_B_ is the Boltzmann constant, and *T* is the absolute temperature. The effective hydrodynamic radius *r*_eff_ can directly be correlated with molecular weight (*M*_w_):
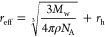
6where ρ is the average protein density (1.37 g/cm^3^) and *N*_A_ is the Avogadro constant. For our calculations, we used a hydration layer radius of 3.2 Å.

Based on studies from the Northeast Structural Genomics Consortium, an empirical formula could be derived for direct correlation of *M*_w_ (in Da) and τ_c_ (in ns) for proteins in the range of 5–25 kDa:^49^
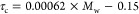
7

The rotational correlation time is directly accessible from the ratio of ^15^N *R*_1_ and *R*_2_ relaxation rates of backbone amide measured at a ^15^N resonance frequency (*v*_N_), assuming slow isotropic overall motion^49,50^ (eq 8):
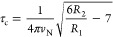
8

All ^19^F NMR spectra were obtained at 298 K on a 600 MHz Bruker Avance III HD NMR spectrometer system equipped with the QCI 600S3 H&F/P/C-N-D-05 Z XT probe. The ^19^F chemical shifts of the inhibitors NJS224 and NJS227 were referenced directly to the signal of TFA (trifluoroacetic acid, −75.48 ppm). 1D ^19^F NMR experiments were recorded with a data size of 2048 complex points, an acquisition time of 36 ms, and 4096 scans per experiment. NJS224 and NJS227 were measured at a concentration of 100 μM in 20 mM Tris pH 8, 150 mM NaCl, 0.5% DMSO, and 10% D2O. Inhibitors were titrated with 20, 50, 100, 200, 300, and 500 μM of each protein construct (*Lp*MIP^1–213^, *Lp*MIP^77–213^, *Lp*MIP^100–213^, *Tc*MIP, and *Bp*MIP). All spectra were processed by using Bruker Topspin 4.0.8.

### Small-Angle X-ray Scattering

SAXS experiments were carried out at the EMBL-P12 bioSAXS beamline, DESY.^51^ Batch mode-SAXS data were collected, *I*(*q*) vs *q*, where *q* = 4πsin *q*/λ is the scattering angle and *l* is the X-ray wavelength (0.124 nm; 10 keV). Data collection was carried out at 20 °C. Automated sample injection and data collection were controlled by BECQUEREL beamline control software.^52^ The SAXS intensities were continuously measured as a series of 0.25 s individual X-ray exposures using a Pilatus 6 M 2D-area detector. The radial averaging of the data to one-dimensional *I*(*q*) vs *q* profiles was carried out with the SASFLOW pipeline incorporating RADAVER from the ATSAS 2.8 software suite.^53^ Profiles were subtracted by probe-free buffer measurements to take account of the buffer’s background scattering. All SAXS data–data comparisons and data-model fits were assessed using the reduced χ^2^ test and the correlation map, or CORMAP, *p*-value.^54^ Fits within the *c*^2^ range of 0.9–1.1 or having CORMAP *p*-values higher than the significance threshold cutoff of *a* = 0.01 are generally considered excellent, i.e., the absence of systematic differences between the data–data or data–model fits at the significance threshold.

Primary SAXS data were analyzed using PRIMUS and additional modules from the ATSAS 3.0.1 software suite.^55^*R*_g_ and the forward scattering at zero angle, *I*(0), were estimated via the Guinier approximation^56^ (ln(*I*(*q*)) vs *q*^2^ for *qR*_g_ < 1.3) and the real-space pair distance distribution function or *p*(*r*) profile. The pair distance distributions were calculated from the indirect inverse Fourier transformation of the data, thus also yielding estimates of the maximum particle dimension, *D*_max_, Porod volume, *V*_p_, shape classification, and concentration-independent molecular weight.^57−59^ Dimensionless Kratky plot representations of the SAXS data (*qR*_g_^2^(*I*(*q*)/*I*(0)) vs *qR*_g_) were generated as previously described.^60^ All collected SAXS data are reported in Table S2.

### Rigid Body Modeling

Rigid-body normal-mode analysis of full-length *Lp*MIP (LpMIP^1–213^) was performed with ATSAS online’s module SREFLEX^38^ using the *Lp*MIP *apo* X-ray crystal structure (PDB: 8BJC) as the template. CRYSOL^61^ was used to assess data-model fits.

### Continuous-Wave EPR Measurements

At the X-band frequency (9.4 GHz), continuous-wave (CW) EPR measurements were conducted using a Bruker EMXnano Benchtop Spectrometer at room temperature. The sample, housed in a 25 μL micropipette (BRAND, Germany) with a 0.64 mm diameter, underwent spectrum recording with the specified parameters: 100 kHz modulation frequency, 0.15 mT modulation amplitude, 0.6–2 mW microwave power, 5.12 ms time constant, 22.5 ms conversion time, and 18 mT sweep width.

### Pulsed EPR Measurements

Pulsed electron paramagnetic resonance (PELDOR/DEER) experiments were performed using a Bruker Elexsys E580 Q-Band (33.7 GHz) Pulsed ESR spectrometer. The experimental setup comprised an arbitrary waveform generator (SpinJet AWG, Bruker), a 50 W solid-state amplifier, a continuous-flow helium cryostat, and a temperature control system (Oxford Instruments). Measurements were conducted at 50 K, employing a 10–20 μL frozen sample containing 15–20% glycerol-d_8_ in a 1.6 mm quartz ESR tube (Suprasil, Wilmad LabGlass).

The measurements for phase memory time (TM) involved utilizing a 48 ns π/2−τ–π Gaussian pulse sequence with a two-step phase cycling, incrementing τ in 4 ns steps. The spectrometer is equipped with a Bruker EN5107D2 dielectric resonator. For PELDOR, a dead-time free four-pulse sequence and a 16-step phase cycling (x[x][xp]x)^62,63^ are employed. A Gaussian pump pulse lasting 38 ns (with a full width at half-maximum (fwhm) of 16.1 ns) is used, alongside a 48 ns observer pulse (fwhm of 20.4 ns). The pump pulse is adjusted to the peak of the echo-detected field-swept spectrum, while the observer pulses are configured to be 80 MHz lower. Deuterium modulations are averaged by gradually increasing the first interpulse delay by 16 ns over 8 steps.

The five-pulse PELDOR/DEER experiments were conducted following the pulse sequence π/2obs – (τ/2 – *t*_0_) – π_pump_ – *t*_0_ – π_obs_ – *t*′ – π_pump_ – (τ – *t*′ + δ) – π_obs_ – (τ_2_ + δ). These experiments were carried out utilizing 48 ns Gaussian observer pulses and a 16-step phase cycling (xxp[x][xp]x) with the same observer pulse settings. For nuclear modulation averaging, a corresponding shift of the standing pump pulse, akin to the 4-pulse PELDOR (16 ns shift in 8 steps), was implemented.

Data analysis for four-pulse experiments utilized Tikhonov regularization implemented in the MATLAB-based DeerAnalysis2019 package.^62^ From the primary data *V*(*t*)/*V*(0), the background (intermolecular interactions *V*(*t*)/*V*(0)) was removed. The obtained form factors *F*(*t*) and *F*(0) were subjected to fitting using a model-free approach to derive distance distributions. To assess the probability distribution error, distances for various background functions were determined by systematically altering the time window and/or the dimensionality for spin distribution (Supporting Information Table S3). Furthermore, the data underwent analysis for distance prediction (and background) in a user-independent manner, employing the deep neural network (DEERNet) analysis integrated into the DeerAnalysis2019 package^62,63^ (Figure S10). The 4-pulse and 5-pulse data were globally analyzed using the Python-based DeerLab program^64^ (Figure S11). Predictions of distance distributions for the structures (PDB 8BJC and 1FD9) were conducted through a rotamer library approach, utilizing the MATLAB-based MMM2022.2 software package.^62^

All synthesized compounds and purified proteins are >95% purity by HPLC analysis and SEC, respectively. Purity of all used proteins was further verified by SDS-PAGE. All chemicals and solvents were procured from authentic commercial sources and used without further purification.

## Data Availability

The X-ray structures of *Tc*MIP in complex with NJS224 and NJS227, as well as *Bp*MIP in complex with NJS227, have been deposited in the PDB under the accession numbers 8P3D, 8P42, and 8P3C. The NMR backbone assignments of *Lp*MIP^1–213^, *Lp*MIP^77–213^, *Bp*MIP, and *Tc*MIP, in complex with NJS224 and NJS227 have been deposited in the BioMagResBank (www.bmrb.io) under the accession numbers 52429, 52430, 52431, 52432, 52433, 52434, 52435, and 52436, respectively. The SAXS data for full-length *Lp*MIP in complex with NJS224 and NJS227 have been deposited in the SASBDB (www.sasbdb.org) under the accession numbers SASDWF4 and SASDWG4, respectively.

## Supporting Information Available

X-ray crystallographic parameters and refinement statistics (Table S1); SAXS data collection and analysis (Table S2); error estimation for EPR measurements (Table S3); characterization of proteins (Figure S1); additional enzymatic (Scheme 1, Figures S2, and S3) and thermal stability assays (Figure S4); and NMR (Figures S6–S9) and EPR (Figures S10 and S11) experiments (PDF)

## Accession Codes

The atomic coordinates and structural factors of *Tc*MIP in complex with NJS224 and NJS227, as well as *Bp*MIP in complex with NJS227, can be found in the RCSB PDB (www.rcsb.org) as entries 8P3D, 8P42, and 8P3C, respectively. The NMR backbone assignments of *Lp*MIP^1–213^, *Lp*MIP^77–213^, *Bp*MIP, and *Tc*MIP, in complex with NJS224 and NJS227, have been deposited in the in the BioMagResBank (www.bmrb.io) under the accession numbers 52429, 52430, 52431, 52432, 52433, 52434, 52435, and 52436, respectively. The SAXS data for full-length *Lp*MIP in complex with NJS224 and NJS227 have been deposited in the SASBDB (www.sasbdb.org) under the accession numbers SASDWF4 and SASDWG4, respectively. Authors will release the atomic coordinates and structure factors upon article publication.

## Abbreviations

*Bp*MIP: *Burkholderia pseudomallei* macrophage infectivity potentiator
CD: circular dichroism
EPR: electron paramagnetic resonance
FKBP: FK506 binding protein
FPA: fluorescence polarization assay
hetNOE: heteronuclear Overhauser effect
*Lp*MIP: *Legionella pneumophila* macrophage infectivity potentiator
NMR: nuclear magnetic resonance
PPIase: peptidyl-prolyl-*cis*–*trans*-isomerase
SAXS: small-angle X-ray scattering
SEC: size-exclusion chromatography
*Tc*MIP: *Trypanosoma cruzi* macrophage infectivity potentiator
