# Supplementary material for: Structure and Dynamics of Macrophage Infectivity Potentiator Proteins from Pathogenic Bacteria and Protozoans Bound to Fluorinated Pipecolic Acid Inhibitors
Source: J Med Chem. 2025 Feb 20;68(5):5926–41. doi: 10.1021/acs.jmedchem.5c00134 (PMC11912469; doi:10.1021/acs.jmedchem.5c00134)
Supplement: Supplementary file 1 — jm5c00134_si_001.pdf [file jm5c00134_si_001.pdf]

“Supporting Information”

Structure and dynamics of macrophage infectivity potentiator proteins from pathogenic bacteria and protozoans bound to fluorinated pipecolic acid inhibitors

Victor Hugo Pérez Carrillo[1, #], Jacob J. Whittaker[2, #], Christoph Wiedemann[1, #], Jean-Martin Harder[1], Theresa Lohr[3], Anil K. Jamithireddy[4], Marina Dajka[5], Benedikt Goretzki[1, 6], Benesh Joseph[5], Albert Guskov[2], Nicholas J. Harmer[4], Ulrike Holzgrabe[3], Ute A. Hellmich[1, 6, 7*]

[1]Faculty of Chemistry and Earth Sciences, Institute of Organic Chemistry and Macromolecular Chemistry, Friedrich Schiller University Jena, 07743 Jena, Germany

[2]Groningen Institute for Biomolecular Sciences and Biotechnology, University of Groningen, 9747AG, Groningen, The Netherlands

[3] Institute of Pharmacy and Food Chemistry, University of Würzburg, Am Hubland, 97074 Würzburg, Germany

[4]Living Systems Institute, University of Exeter, Stocker Road, Exeter EX4 4QD, UK

[5]Department of Physics, Free University of Berlin, 14195 Berlin, Germany

[6]Center for Biomolecular Magnetic Resonance, Goethe-University, 60438 Frankfurt/Main, Germany

[7]Cluster of Excellence “Balance of the Microverse”, Friedrich Schiller University Jena, 07743 Jena, Germany

*Correspondence to UAH: ute.hellmich@uni-jena.de

[#]These authors contributed equally: V.H.P.C., J.J.W., C.W.

Table of Contents - Supporting Information

Table S1: X-ray crystallography data collection and refinement statistics (molecular replacement)

Table S2: SAXS data reporting table for full-length *Lp*MIP

Table S3: Error estimation for EPR measurements

Scheme S1: Tracer molecule NJS254

Fig. S1: Structural comparison and purification of MIP proteins from various pathogens

Fig S2. Enzymatic activity of MIP orthologs

Fig. S3: Fluorescence polarization assay (FPA)

Fig. S4: Thermal stability of microbial MIP proteins

Fig. S5: X-ray crystallography of *Tc*MIP and *Bp*MIP in the apo and inhibitor bound states

Fig. S6: Backbone NMR assignments of *Trypanosma cruzi* MIP constructs in the apo and inhibitor bound states

Fig. S7: Backbone NMR assignments of *Burkholderia pseudomallei* MIP constructs in the apo and inhibitor bound states

Fig. S8: Backbone NMR assignments of *Legionella pneumophila MIP* constructs in the apo and inhibitor bound states

Fig. S9: ^{19}F NMR spectra of inhibitors NJS224 and NJS227 titrated with MIP proteins

Fig. S10: PELDOR/DEER data analysis for spin-labeled *Lp*MIP K80C

Fig S11: PELDOR/DEER data analysis for *Lp*MIP S208C

Table S1: X-ray crystallography data collection and refinement statistics (molecular replacement).

*Values in parentheses are for the highest resolution shell.

	***Tc*MIP + NJS224**	***Tc*MIP + NJS227**	***Bp*MIP + NJS227**
**PDB-ID**	**8P3D**	**8P42**	**8P3C**
Wavelength	0.9763	0.9677	0.9677
Resolution range	46.77 - 1.71 (1.771 - 1.71)	32.41 - 2.643 (2.738 - 2.643)	41.61 - 2.02 (2.092 - 2.02)
Space group	*P* 1 21 1	*P* 41	*P* 31 2 1
a, *b*, *c* (Å)	46.86 34.70 53.08	87.74 87.74 57.51	77.608 77.608 52.995
α, β, γ (°)	90, 93.53, 90	90, 90, 90	90 90 120
Unique reflections	18060 (1811)	12330 (1225)	12368 (1233)
Completeness (%)	96.58 (98.00)	94.84 (93.94)	99.76 (99.84)
Mean I/sigma(I)	17.5 (3.33)	12.6 (2.80)	15.11 (3.04)
Wilson B-factor	25.99	39.81	30.77
R-meas	0.04	0.07	0.04
CC1/2	96.3(28.0)	94.20 (30.05)	96.54 (28.48)
Reflections used in refinement	18058 (1811)	12325 (1225)	12365 (1234)
Reflections used for R-free	860 (83)	649 (68)	522 (45)
R-work	0.1831 (0.2335)	0.2246 (0.3357)	0.2254 (0.2867)
R-free	0.2211 (0.2978)	0.2715 (0.3101)	0.2574 (0.3337)
Number of non-hydrogen atoms	1499	2730	1103
macromolecules	1292	2514	964
ligands	72	145	58
solvent	168	71	81
Protein residues	162	316	126
RMS(bonds) / Å	0.442	0.662	0.674
RMS(angles) / °	6.36	8.03	7.82
Ramachandran favored (%)	98.75	96.79	96.77
Ramachandran allowed (%)	1.25	2.88	1.61
Ramachandran outliers (%)	0.00	0.32	1.61
Rotamer outliers (%)	0.75	3.46	6.00
Clashscore	2.69	7.30	6.07
Average B-factor	30.58	37.50	35.70
macromolecules	29.89	37.55	35.62
ligands	27.03	37.23	33.15
solvent	36.72	36.08	38.50

*Values in parentheses are for the highest resolution shell.

Table S2: SAXS data reporting table for full-length *Lp*MIP.

**Sample details**		
SAMPLE	*Lp*MIP + NJS224	*Lp*MIP + NJS227
SASBDB Accession Codes	SASDWF4	SASDWF4
Organism	*Legionella pneumophilia*	
NCBI protein accession ID	66489975	
(amino acid range)	1-213*	
SAXS buffer	20 mM Tris pH 7.5	
NaCl concentration	150 mM	
Sample injection volume	45	
Sample injection conc.	7 mg/mL	5 mg/mL
**Instrument details**		
Instrument	EMBL P12 bioSAXS beam line, DESY, Hamburg	
Exposure time/# frames	0.10 s (27)	0.10 s (11)
X-ray wavelength/energy	0.124 nm (10000.2 eV)	
Sample-to-detector distance	3 m	
Scattering intensity scale	Arbitrary unit, a.u.	
Working s-range (nm^{-1})	0.02-7.37	0.02-7.37
**Guinier analysis:**		
Primary data analysis software	PRIMUS (ATSAS 3.0.1)	
Guinier I(0) (σ)	0.23(<0.01)	0.17(<0.01)
R_g (Guinier, nm) (σ)	2.93(<0.01)	2.95(0.01)
sR_g range	0.22-1.30	0.32-1.28
**p(r) analysis:**		
Method	GNOM 5	
I(0), POR (σ)	0.23(<0.01)	0.17(<0.01)
R_g (POR, nm) (σ)	2.96(<0.01)	3.00(<0.01)
D_{max} (nm)	9.74	10.1
Quality of fit, CorMap P / χ^2	0.61 / 1.10	0.37 / 1.18
Porod volume (nm^3)	58	65
Shape classification	globular	globular
**Molecular Weight analysis:**		
MW, calculated from amino acid sequence, kDa	46.5 (dimer with 2x NJS224)	46.5 (dimer with 2x NJS227)
MW from SAXS data, kDa	40.6-44.3	34.2-39.8
**Rigid body/Normal mode modelling:**		
Method	SREFLEX (five individual fits)	
Symmetry	P1	P1
Template	8BJC	8BJC
Initial template fit, CorMap P / χ^2	1.89E-129 / 88.87	5.07E-77 / 30.07
	4.2E-04 / 1.29	5.78E-01 / 1.15
	2.0E-06 / 1.50	3.49E-01/ 1.16
Final model fit, CorMap P / χ^2	3.95E-10 / 1.57	5.78E-01 / 1.17
	4.07E-07 / 1.61	2.64E-02 / 1.19
	9.03E-20 / 1.63	3.49E-01 / 1.21

*The nomenclature for *Lp*MIP$^{1\text{-}213}$ used in this manuscript refers to the processed protein after cleavage of the N-terminal signal peptide comprising residues 1-20, which would be denoted as *Lp*MIP$^{21\text{-}233}$ according to the UniProt protein accession ID.

Table S3: Error estimation for EPR measurements. Parameters for error estimation of the probability distributions obtained using Tikhonov regularization for full-length *Lp*MIP K80C variant (see Fig. 5, S9). Validation was performed as featured in the DeerAnalysis2019 software package (1)

**Sample**	**Error validation**					**Regularization parameter (α)**
	dimensionality (*d*)		starting time window			
	range	steps	range (ns)	steps	T_{max} (µs)	
**K80C**	2.8-3.2	9	240-1000	11	5.36	1258
**K80C + NJJ224**	2.8-3.2	9	240-1000	11	4.40	1258
**K80C + NJJ227**	2.8-3.2	9	240-1000	11	4.50	1000

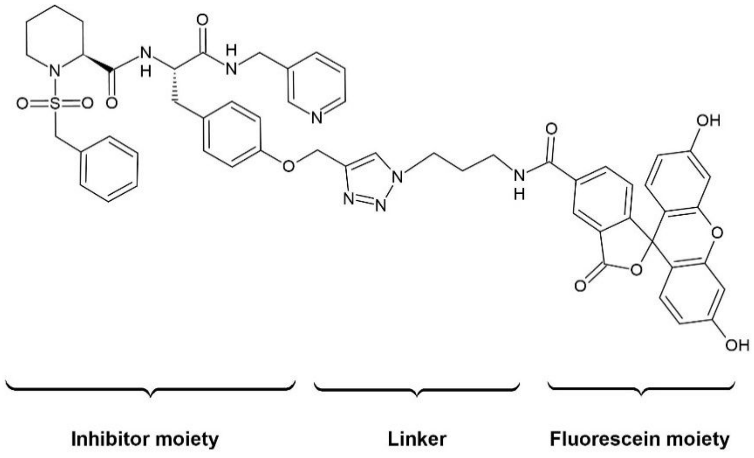

Scheme S1: Tracer molecule NJS254 was developed specifically for the Fluorescent Polarization Assay (FPA) (2) (compare Table 1, main manuscript). NJS254 is composed of a MIP-based-inhibitor and a fluorescein moiety covalently attached via a triazole linker.

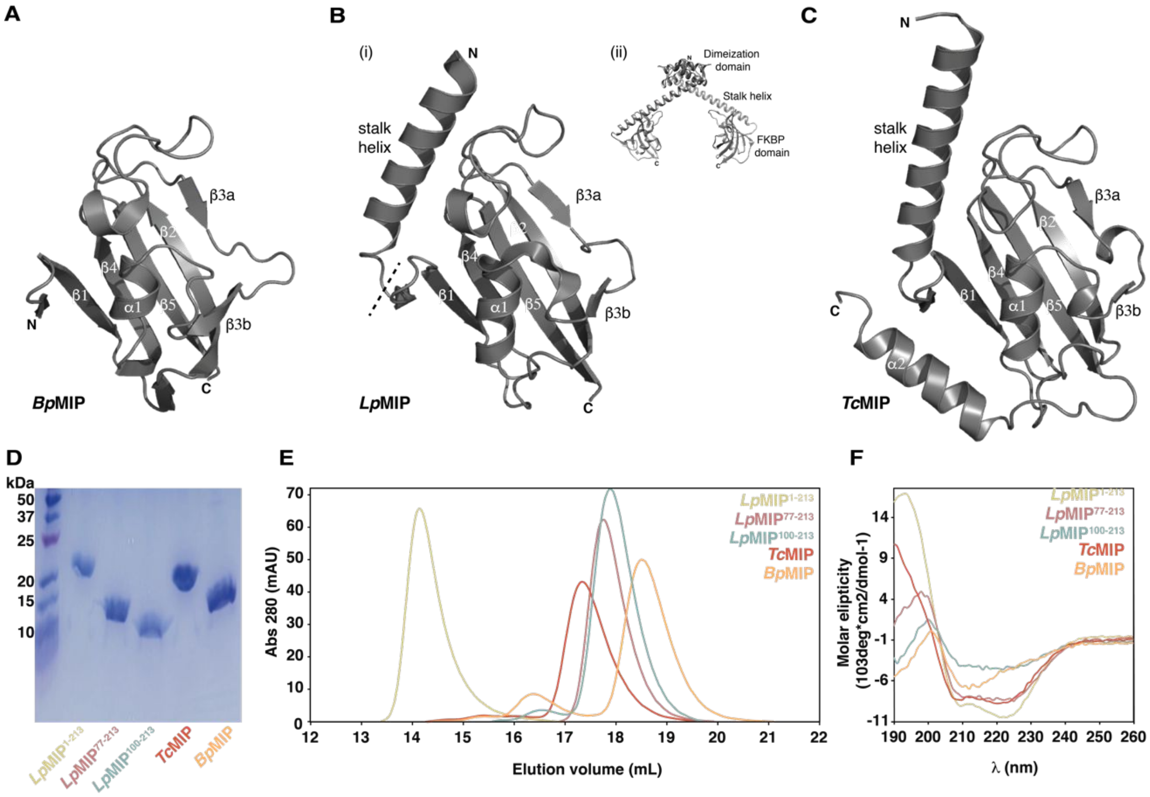

Fig. S1: Structural comparison and purification of MIP proteins from various pathogens. (A-C) Structures of full-length *Burkholderia pseudomallei* MIP (*Bp*MIP) (PDB: 8P3C), *Legionella pneumophila* MIP (*Lp*MIP) variants and full-length *Trypanosoma cruzi* MIP (*Tc*MIP) (PDB: 8P3D) (4). The respective β-strands and α-helices are marked in all proteins. For *Lp*MIP, an N-terminal deletion construct (i) from amino acid 77-213 (LpMIP$^{77\text{-}213}$) featuring half of the stalk helix is shown. The dashed line indicates the N-terminus for construct LpMIP$^{100\text{-}213}$. Full-length *Lp*MIP is the only homodimeric MIP (PDBs: 8BK5, 8BJC) used for this study (ii) and contains an N-terminal dimerization domain absent in BpMIP and TcMIP (14). Note that the stalk helix, which is not present in native *Bp*MIP, is marked "stalk" to align the respective secondary structure elements across *Lp*MIP and *Tc*MIP proteins. *Tc*MIP contains an additional C-terminal α-helix (α2) not present in *Bp*MIP and *Lp*MIP. In all three proteins, the β4/β5 loop is the longest loop and shows the largest structural variations across MIP protein structures determined to date. (D) SDS-PAGE of purified MIP proteins from *Legionella pneumophila* (*Lp*MIP), *Trypanosoma cruzi* (*Tc*MIP) and *Burkholderia pseudomallei* (*Bp*MIP). Three different constructs of *Lp*MIP, i.e. the full-length protein *Lp*MIP $^{1\text{-}213}$, an N-terminal truncation leaving half of the stalk helix in place (*Lp*MIP$^{77\text{-}213}$ and an N-terminal truncation leaving only the FKBP-like domain (*Lp*MIP$^{100\text{-}213}$) were included in the analysis, as these constructs resemble the domain architecture of *Tc*MIP and *Bp*MIP, respectively. (E) Analytical size exclusion chromatography of MIP constructs. Note that full-length *Lp*MIP is a homodimer, while all shorter MIP proteins are monomeric. (F) Circular dichroism spectroscopy of purified MIP constructs.

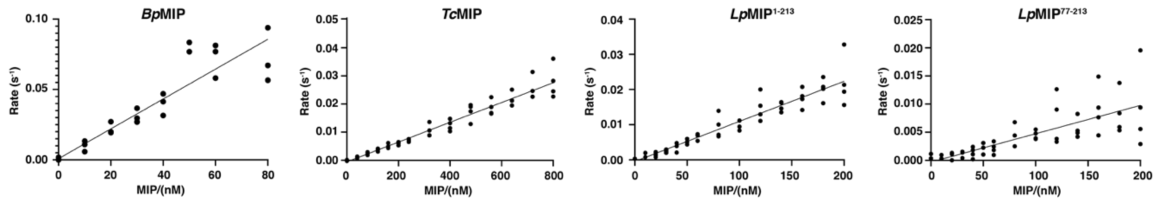

Fig S2. Enzymatic activity of MIP orthologs. The activity of the MIPs from *B. pseudomallei*, *T. cruzi*, and *L. pneumophila* was determined using a standard coupled assay with chymotrypsin (Table 1). Results are reflective of at least two replicates conducted on different days. Activity (as well as inhibition) of *Lp*MIP$^{100\text{-}213}$ could not be determined reliably as its basal activity was on the low end of the detection limit of the assay (see also Table 1, main manuscript).

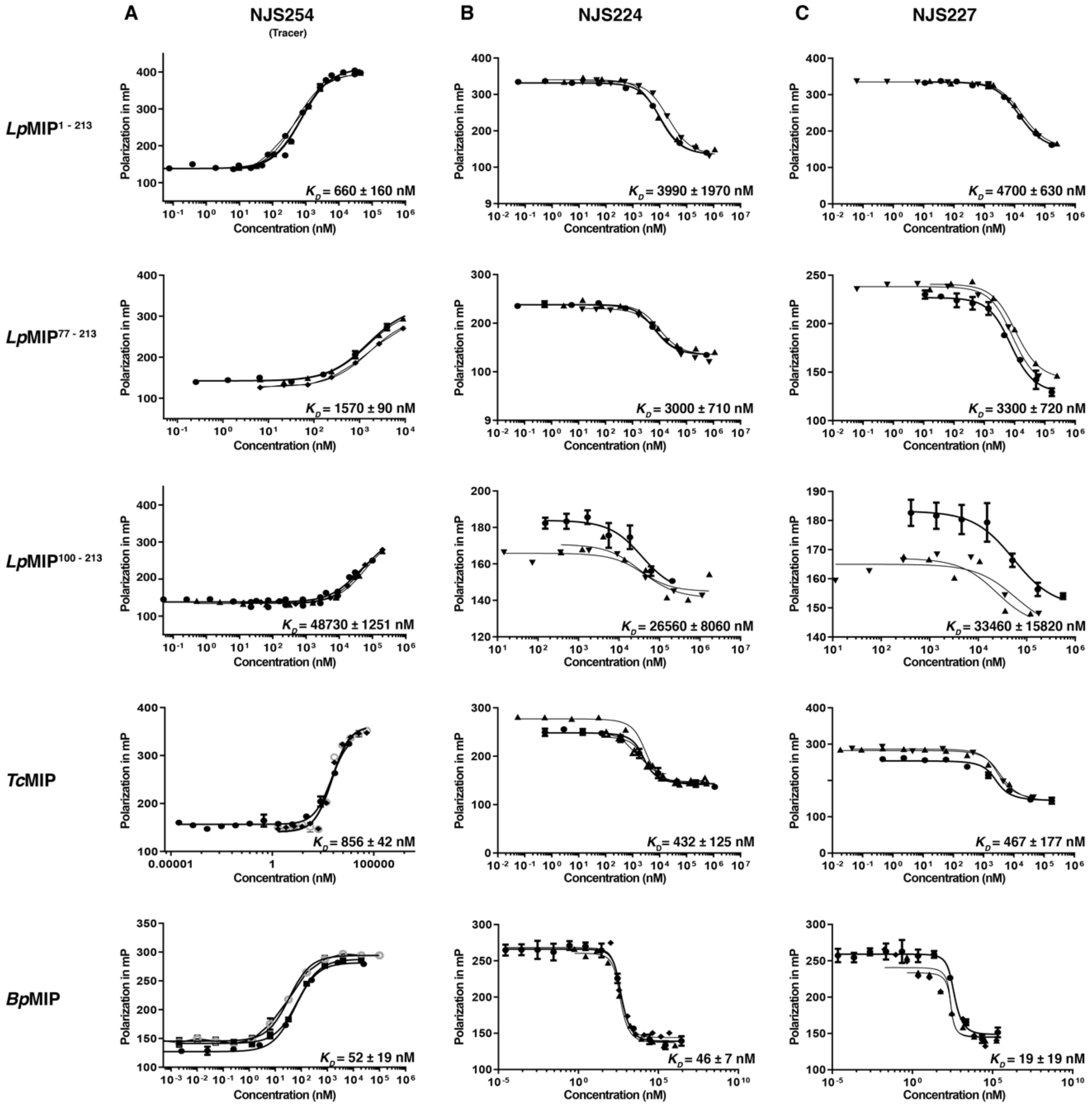

Fig. S3: Fluorescence polarization assay (FPA). Using the tracer molecule NJS254 (2) (Scheme S1), the interaction of various *L. pneumophila* MIP constructs as well as full-length *B. pseudomallei* and *T. cruzi* MIP with inhibitors NJS224 and NJS227 was investigated to yield (A) the K_D of the tracer molecule as well as the respective K_i values for (B) NJS224 and (C) NJS227 through the displacement of the fluorescent tracer from the respective target protein. All experiments were carried out in technical replicates, SEM are from n=3.

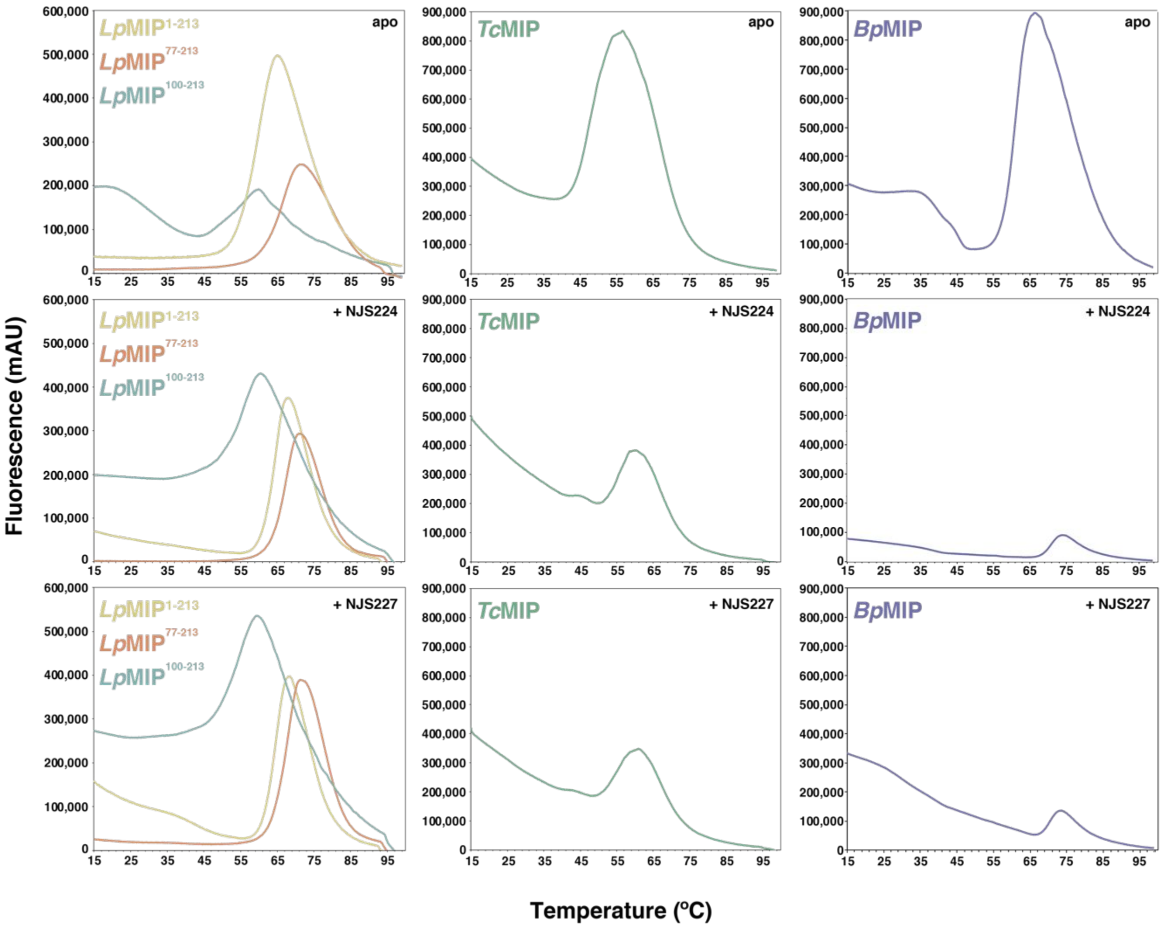

	T_m (°C) apo	T_m (°C) + NJS224	T_m (°C) + NJS227
*Lp*MIP$^{1\text{-}213}$	58.8 ± 0.3	62.9 ± 1.1	63.3 ± 0.3
*Lp*MIP$^{77\text{-}213}$	65.1 ± 1.3	66.9 ± 0.9	66.8 ± 0.2
*Lp*MIP$^{100\text{-}213}$	52.6 ± 1.3	54.5 ± 1.1	53.5 ± 0.5
*Tc*MIP	47.9 ± 0.6	55.3 ± 0.2	55.0 ± 1.6
*Bp*MIP	61.6 ± 0.1	69.6 ± 0.1	68.5 ± 0.6

Fig. S4: Thermal stability of microbial MIP proteins. The melting temperatures (T_m) for different MIP constructs in the absence (apo) or in the presence of five-fold excess of NJS224 or NJS227 were determined with a fluorescence-based melting assay. Experiments were carried out in technical replicates, SEM are from n=3.

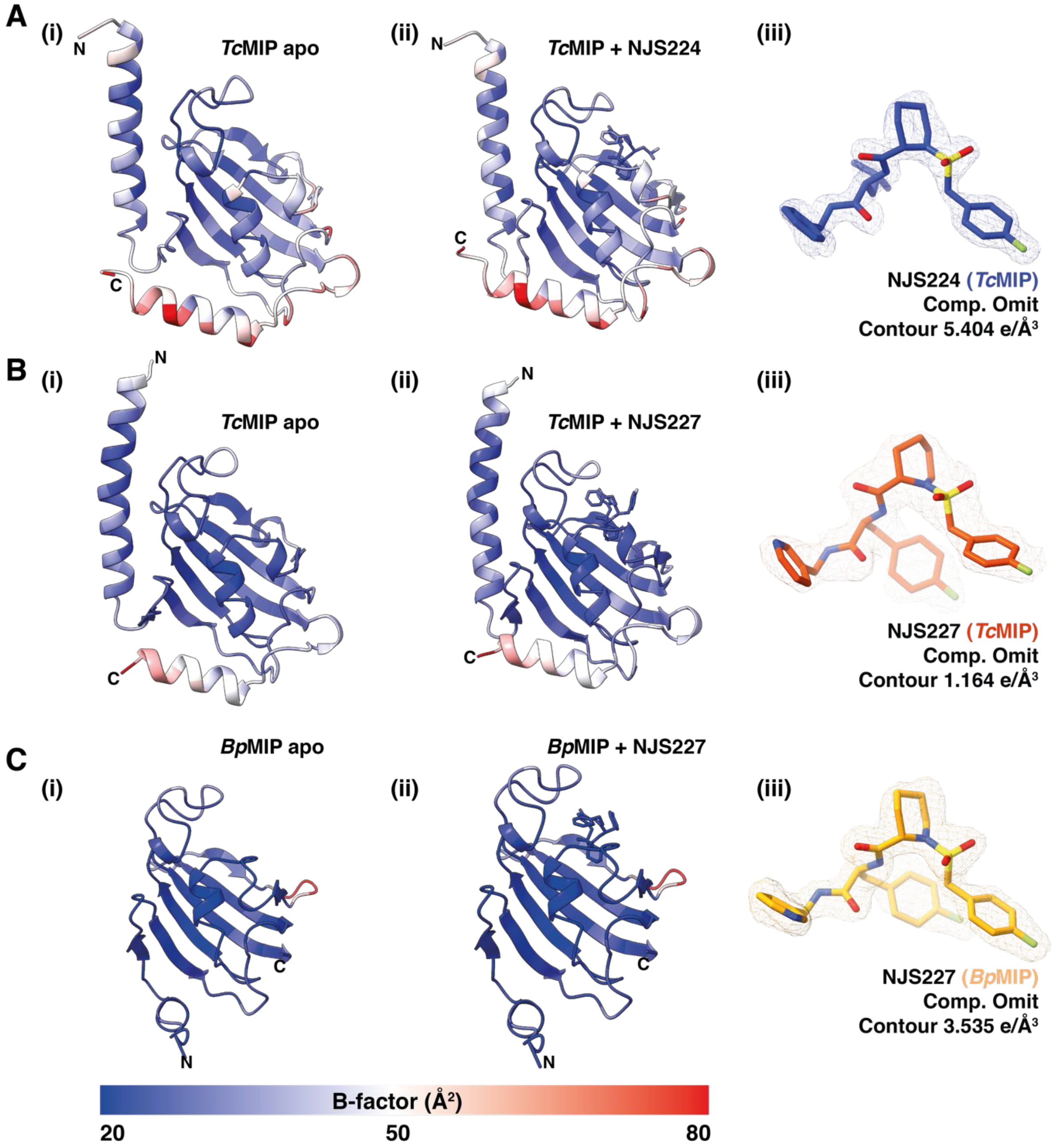

Fig. S5: X-ray crystallography of *Tc*MIP and *Bp*MIP in the apo and inhibitor bound states. Crystallographic B-factors in the absence (i) and presence (ii) of ligands for *Tc*MIP (A, B) and *Bp*MIP (C). Composite omit maps of NJS inhibitors bound to *Tc*MIP (A,B) and *Bp*MIP (C) are shown in (iii).

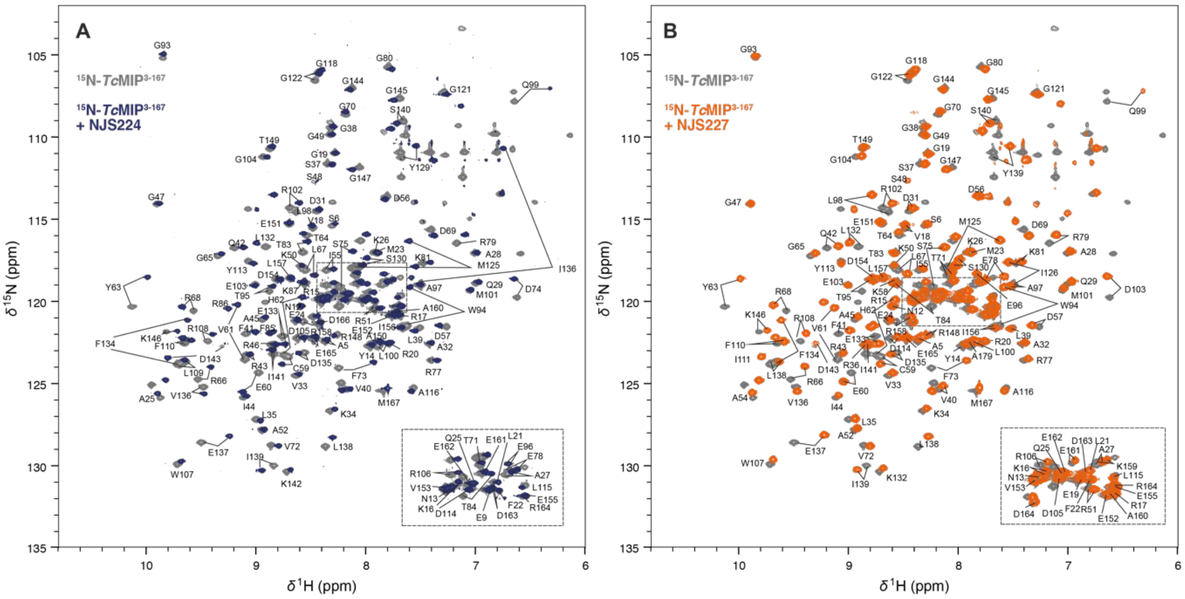

Fig. S6: Backbone NMR assignments of *Trypanosma cruzi* MIP constructs in the apo and inhibitor bound states. Backbone assignments of *Tc*MIP without (grey) and with 5-fold molar excess of (A) NJS224 (orange) or (B) NJS227 (blue). Previously published backbone amide resonance assignments for apo *Tc*MIP (BMRB entry 27531) (4) could be transferred to our *Tc*MIP spectra in the apo state. Backbone assignments of *Tc*MIP in the NJS224 (BMRB entry 52436) and NJS227 (BMRB entry 52435) bound state were determined using 3D assignment experiments under our buffer conditions.

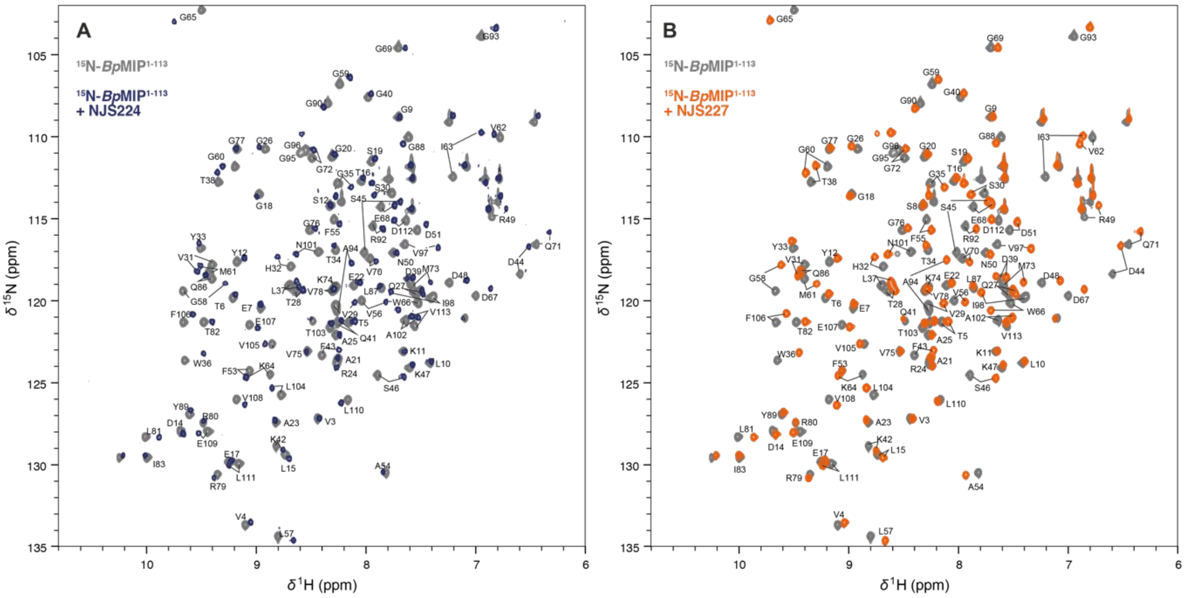

Fig. S7: Backbone NMR assignments of *Burkholderia pseudomallei* MIP constructs in the apo and inhibitor bound states. Backbone assignments of *Bp*MIP without (grey) and with 5-fold molar excess of (A) NJS224 (orange) or (B) NJS227 (blue). Previously published backbone amide resonance assignments for *Bp*MIP (BMRB entry 16406 and 17151) could be transferred to our *Bp*MIP spectra in the apo state. Backbone assignments of *Bp*MIP in the NJS224 (BMRB entry 52433) and NJS227 (BMRB entry 52434) bound state were determined using 3D assignment experiments under our buffer conditions.

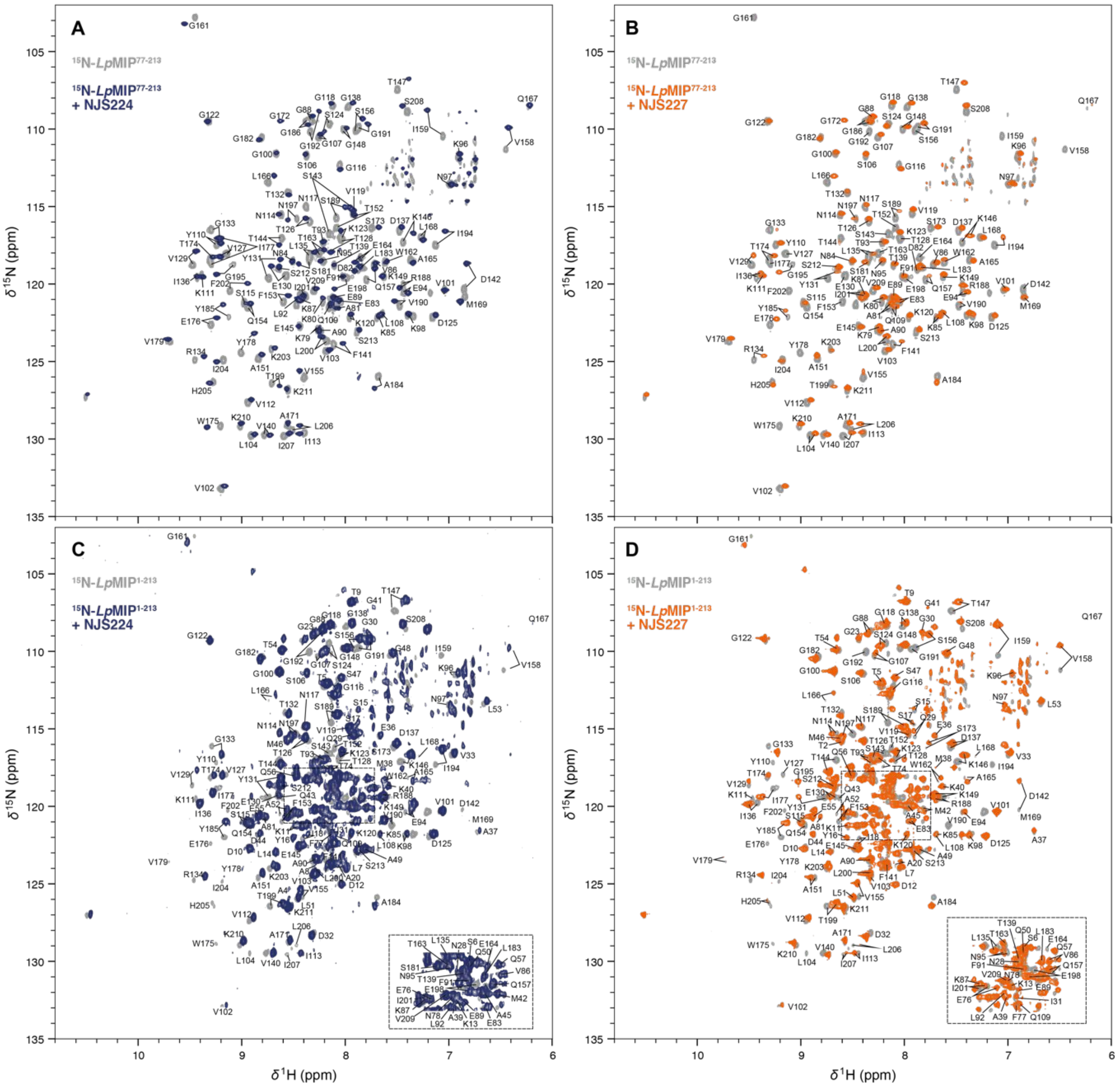

Fig. S8: Backbone NMR assignments of *Legionella pneumophila* MIP constructs in the apo and inhibitor bound states. (A, B) Backbone assignments of *Lp*MIP$^{77\text{-}213}$ without (grey) and with 5-fold molar excess of (A) NJS224 (orange) or (B) NJS227 (blue). (C, D) Backbone assignments of full-length *Lp*MIP$^{1\text{-}213}$ without (grey) and with 5-fold molar excess of (C) NJS224 (orange, BMRB entry 52429) or (D) NJS227 (blue, BMRB entry 52430). Previously published backbone amide resonance assignments for *Lp*MIP$^{1\text{-}213}$ (BMRB entry 7021) (5) and *Lp*MIP$^{77\text{-}213}$ (BMRB entry 6334) (6) could be transferred to our *Lp*MIP$^{1\text{-}213}$ and *Lp*MIP$^{77\text{-}213}$ spectra, respectively, in the apo state. Backbone assignments of *Lp*MIP$^{77\text{-}213}$ in the NJS224 (BMRB entry 52432) and NJS227 (BMRB entry 52431) bound state were determined using 3D assignment experiments under our buffer conditions.

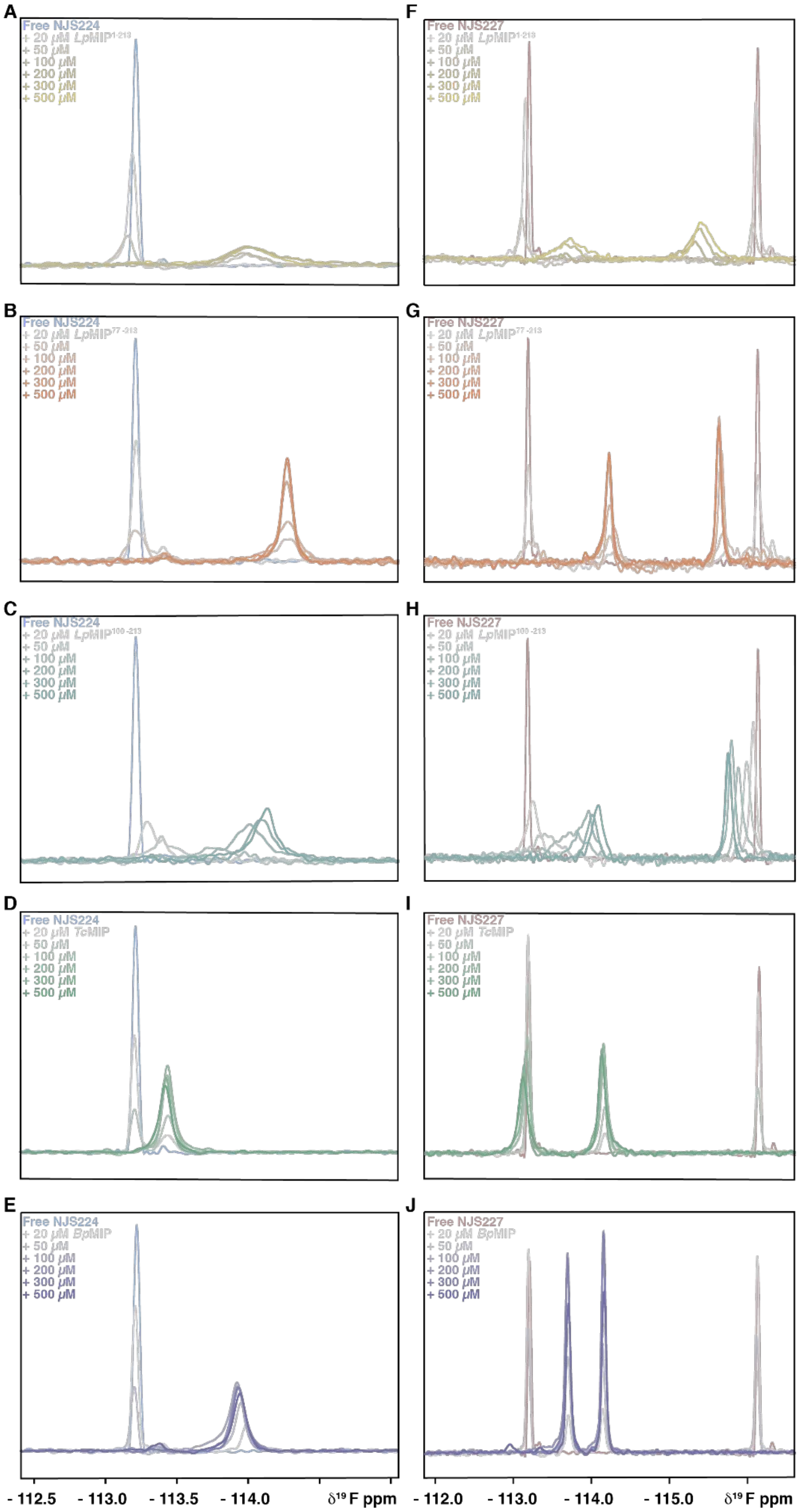

Fig. S9: ¹⁹F NMR spectra of inhibitors NJS224 and NJS227 titrated with MIP proteins. (A-E) Titration of NJS224 with different concentration of purified *Lp*MIP$^{1\text{-}213}$ (full-length, dimer), *Lp*MIP$^{77\text{-}213}$, *Lp*MIP$^{100\text{-}213}$, *Tc*MIP and *Bp*MIP, respectively. **(F-J)** Titration of NJS227 with different concentration of *Lp*MIP$^{1\text{-}213}$ (full-length, dimer), *Lp*MIP$^{77\text{-}213}$, *Lp*MIP$^{100\text{-}213}$, *Tc*MIP and *Bp*MIP respectively.

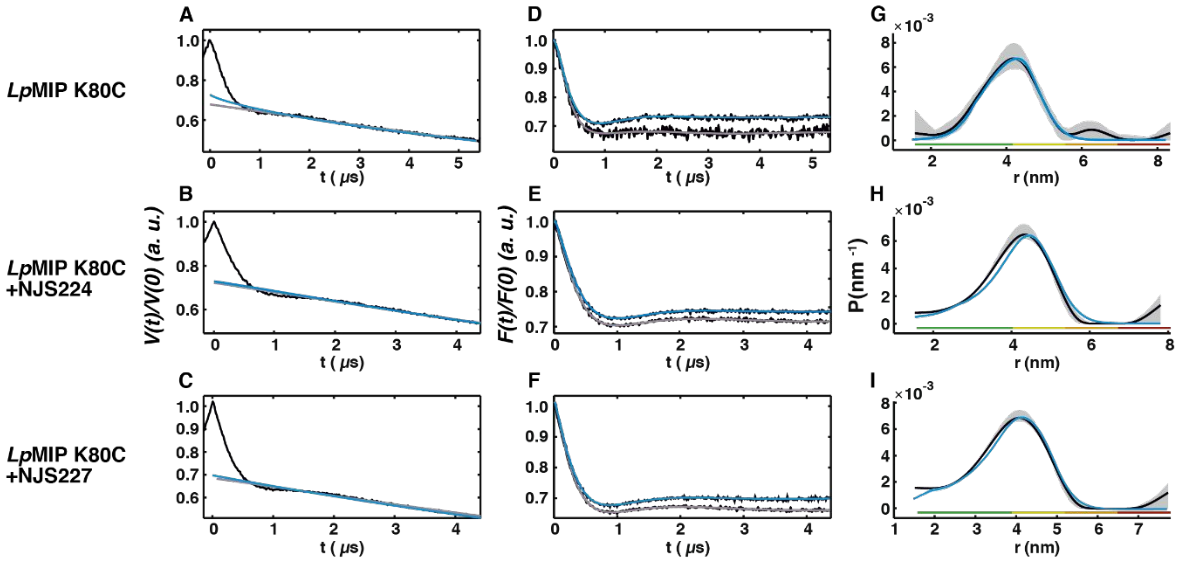

Fig. S10: PELDOR/DEER data analysis for spin-labeled *Lp*MIP K80C. (A, B, C) The primary data (black) overlaid with the intermolecular (background) contribution from deep neural network analysis (blue) and Tikhonov regularization (grey). (D, E, F) The background corrected form factors overlaid with the fits. (G, H, I) The corresponding distance distributions. *Lp*MIP K80C was incubated with 5-fold molar excess of NJS224 or NJS227.

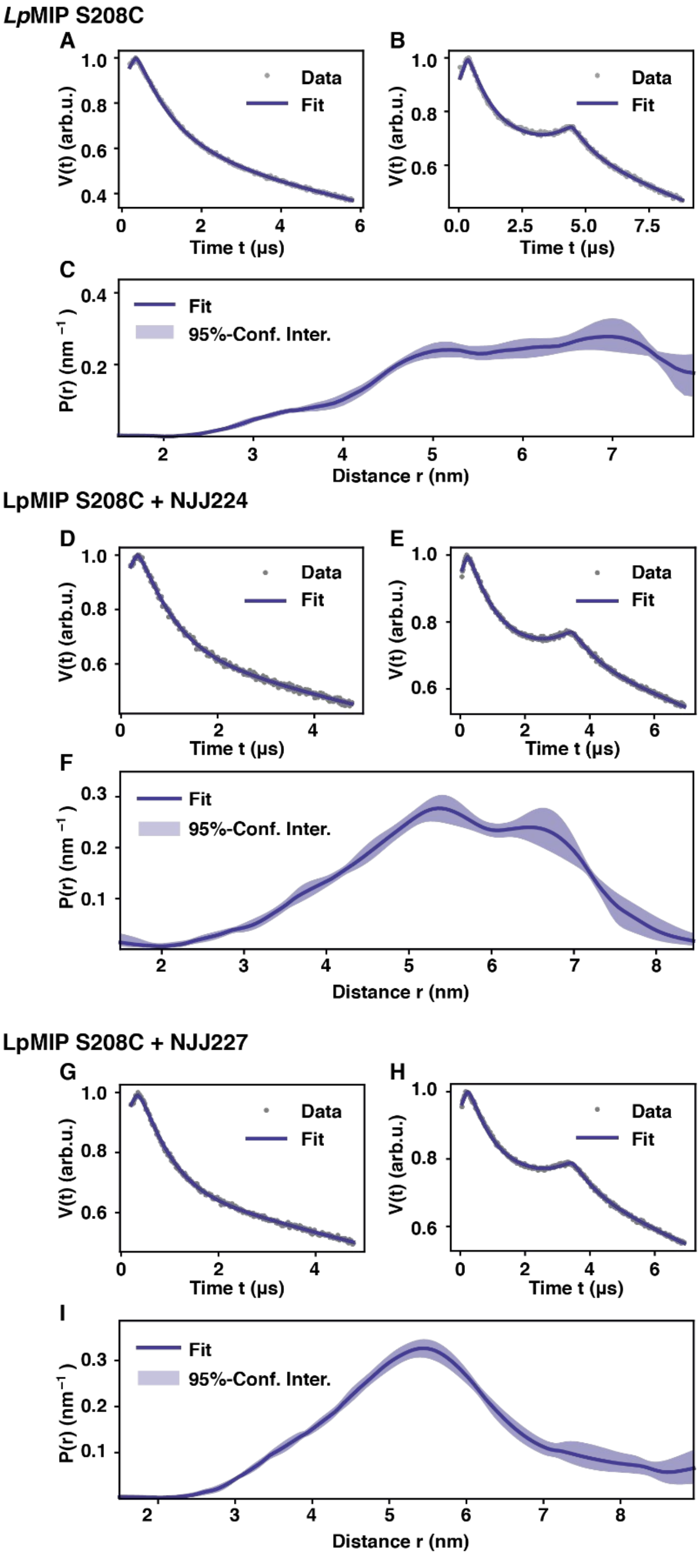

Fig S11: PELDOR/DEER data analysis for *Lp*MIP S208C. The 4-pulse and 5-pulse PELDOR data of apo or inhibitor-bound, spin-labelled *Lp*MIP S208C were globally analyzed using the Python based DeerLab program. (A, D, G) The 4-pulse PELDOR data (grey) is overlaid with the fit (blue). (B, E, H) The 5-pulse PELDOR data (grey) overlaid with the fit (blue); (C, F, I) The corresponding distance distributions with a 95% confidence interval (shaded in light blue). *Lp*MIP S280C was incubated with 5-fold molar excess of NJS224 or NJS227.

References for Supporting Information

1- Polyhach Y, Bordignon E, Jeschke G. Rotamer libraries of spin labelled cysteines for protein studies. Phys Chem Chem Phys. 2011;13(6):2356–66.

2- Scheuplein NJ, Bzdyl NM, Lohr T, Kibble EA, Hasenkopf A, Herbst C, et al. Analysis of Structure–Activity Relationships of Novel Inhibitors of the Macrophage Infectivity Potentiator (Mip) Proteins of *Neisseria meningitidis*, *Neisseria gonorrhoeae*, and *Burkholderia pseudomallei*. J Med Chem. 2023 Jul 13;66(13):8876–95.

3- Wiedemann C, Whittaker JJ, Pérez Carrillo VH, Goretzki B, Dajka M, Tebbe F, et al. Legionella pneumophila macrophage infectivity potentiator protein appendage domains modulate protein dynamics and inhibitor binding. Int J Biol Macromol. 2023 Dec; 252:126366.

4- Lopez JM, Antiparra R, lippens G, Zimic M, Sheen P, Maruenda H. Backbone chemical shift assignment of macrophage infectivity potentiator virulence factor of Trypanosoma cruzi. Biomol NMR Assign. 2019 Apr 18;13(1):21–5.

5- Horstmann M, Ehses P, Schweimer K, Steinert M, Kamphausen T, Fischer G, et al. Domain Motions of the Mip Protein from *Legionella pneumophila*. Biochemistry. 2006 Oct 1;45(40):12303–11.

6- Horstmann M, Kamphausen T, Schweimer K, Steinert M, Hacker J, Haase A, et al. Letter to the Editor: 1H, 13C, 15N backbone and sidechain resonance assignment of Mip (77-213) the PPIase domain of the Legionella pneumophila Mip protein. J Biomol NMR. 2005 Jan;31(1):77–8.
